# Genetic variants in root architecture-related genes in a *Glycine soja* accession, a potential resource to improve cultivated soybean

**DOI:** 10.1186/s12864-015-1334-6

**Published:** 2015-02-25

**Authors:** Silvas J Prince, Li Song, Dan Qiu, Joao V Maldonado dos Santos, Chenglin Chai, Trupti Joshi, Gunvant Patil, Babu Valliyodan, Tri D Vuong, Mackensie Murphy, Konstantinos Krampis, Dominic M Tucker, Ruslan Biyashev, Anne E Dorrance, MA Saghai Maroof, Dong Xu, J Grover Shannon, Henry T Nguyen

**Affiliations:** National Center for Soybean Biotechnology and Division of Plant Sciences, University of Missouri, Columbia, MO 65211 USA; Christopher S. Bond Life Sciences Center, University of Missouri, Columbia, MO 65211 USA; Department of Computer Science, University of Missouri, Columbia, MO 65211 USA; Department of Crop and Soil Environmental Sciences, Virginia Tech, Blacksburg, VA 24061 USA; Department of Plant Pathology, The Ohio State University, OARDC, Wooster, OH 44691 USA

**Keywords:** Root, Quantitative trait locus, Soybean, Wild soybean, Root architecture, Non-synonymous SNP, Microarray, Single feature polymorphism, DNA sequencing

## Abstract

**Background:**

Root system architecture is important for water acquisition and nutrient acquisition for all crops. In soybean breeding programs, wild soybean alleles have been used successfully to enhance yield and seed composition traits, but have never been investigated to improve root system architecture. Therefore, in this study, high-density single-feature polymorphic markers and simple sequence repeats were used to map quantitative trait loci (QTLs) governing root system architecture in an inter-specific soybean mapping population developed from a cross between *Glycine max* and *Glycine soja.*

**Results:**

Wild and cultivated soybean both contributed alleles towards significant additive large effect QTLs on chromosome 6 and 7 for a longer total root length and root distribution, respectively. Epistatic effect QTLs were also identified for taproot length, average diameter, and root distribution. These root traits will influence the water and nutrient uptake in soybean. Two cell division-related genes (*D type cyclin* and *auxin efflux carrier protein)* with insertion/deletion variations might contribute to the shorter root phenotypes observed in *G. soja* compared with cultivated soybean. Based on the location of the QTLs and sequence information from a second *G. soja* accession, three genes (*slow anion channel associated 1 like*, *Auxin responsive NEDD8-activating complex* and *peroxidase*), each with a non-synonymous single nucleotide polymorphism mutation were identified, which may also contribute to changes in root architecture in the cultivated soybean. In addition, *Apoptosis inhibitor 5-like* on chromosome 7 and *slow anion channel associated 1-like* on chromosome 15 had epistatic interactions for taproot length QTLs in soybean.

**Conclusion:**

Rare alleles from a *G. soja* accession are expected to enhance our understanding of the genetic components involved in root architecture traits, and could be combined to improve root system and drought adaptation in soybean.

**Electronic supplementary material:**

The online version of this article (doi:10.1186/s12864-015-1334-6) contains supplementary material, which is available to authorized users.

## Background

*Glycine soja*, the annual wild progenitor of cultivated soybean, is widely distributed among East Asian countries. In China, the cultivated soybean was domesticated from wild soybean more than 5,000 years ago [1] and underwent two rounds of whole genome duplication [2]. The first genome duplication occurred within the last 60 million years and the latter between 5 and 13 million years ago. Both *G. soja* and *Glycine max* have prominent differences for various morphological and physiological characters, known as domestication syndrome [3]. In soybean, the process of plant breeding accelerated genetic gain and narrowed the genetic base [4]. The genetic diversity among 99% of North American cultivars released between 1947 and 1988 could be traced back to only 0.02% of the landraces [1]. This loss in diversity among high-yielding adapted lines ultimately inhibits future genetic gains in productivity, broadens susceptibility to new pests and diseases, and acts as a threat to food security [4]. In contrast to modern soybean cultivars, wild soybeans are genetically diverse, with valuable rare alleles [5]. Recent advances in sequencing technologies also highlighted the uniqueness of genomic content in both cultivated and wild soybean, and provide an opportunity to use *G. soja* to broaden the genetic base of cultivated soybean [6,7]. In addition, assessing genomic differences for key traits will provide insights into the process of speciation and domestication, and will deepen our understanding of the origin of genes involved in complex traits [8].

Earlier studies showed that the presence of unique alleles in wild/weedy species and primitive land races could be used to improve agronomic traits in crop plants [9]. Later, alleles were successfully introgressed from wild species and deployed in different crops through genetic mapping and molecular marker approaches [9,10]. A number of array-based high-throughput marker genotyping platforms have been used in plant breeding, especially marker-assisted selection, to understand crop domestication and plant evolution [11]. These microarray-based markers have been used for high-density molecular map construction, quantitative trait locus (QTL)/expression QTL mapping, and genetic diversity analysis [11]. Among these array-based markers, single-feature polymorphism (SFP) was originally used for fine mapping and positional cloning of genes in yeast [12]. Later, it was used in plant species with both small and complex genomes [11]. SFPs have been widely used for different applications, such as molecular linkage map construction and QTL mapping in *Arabidopsis* [13], as well as in major cereal crops [14] and legumes [15].

The effective use of wild relatives to improve a wide variety of traits from yield to stress tolerance in cultivated/domesticated crops was reviewed [16] and has been successfully applied in rice [17] and wheat [18]. Similarly, inter-specific variation in soybean was used to identify novel alleles in *G. soja* that influence various traits, including domestication [19], alkaline and salt tolerance [20], dehydration tolerance [21], yield [22], resistance to pathogens and pests, and seed compositional traits [23]. Among abiotic stresses, drought stress causes tremendous yield losses in soybean [24]. Drought avoidance is considered to be the most relevant process to mitigate agricultural drought and maintain crop performance [25]. Root system architecture (RSA) and root hydraulics are the key traits that affect water capture under drought-prone environments [26,27] and sustain yield in sub-optimal conditions. Thus, RSA and root distribution within the environment are important to understand nutrient and water use efficiency in plants [28]. Recent studies in rice have shown that an increase in root depth leads to an increase in water uptake, which is translated into higher grain yield under rain-fed conditions [29]. The existence of genetic variation for root growth and architecture within various crop species makes RSA a promising target for crop improvement programs [30]. A recent study of inter-specific tomato introgression lines also emphasized the need to identify genes associated with favorable root traits and their transcription regulation [31]. To the best of our knowledge, *G. soja* alleles have never been used to improve root system architecture. This is understandable because *G. soja* roots are often very thin, with narrow hairs, as well as reduced root mass and volume. Thus, the objectives of the present study were (i) to identify novel alleles from a *G. soja* accession to explore the possibility of enhancing root architectural traits in cultivated soybean; (ii) to detect significant QTL regions and identify candidate genes governing root traits, and (iii) to understand the mechanisms regulating the transcript variation in an inter-specific mapping population.

## Results

### Phenotypic variation of root traits

**Figure 1:**
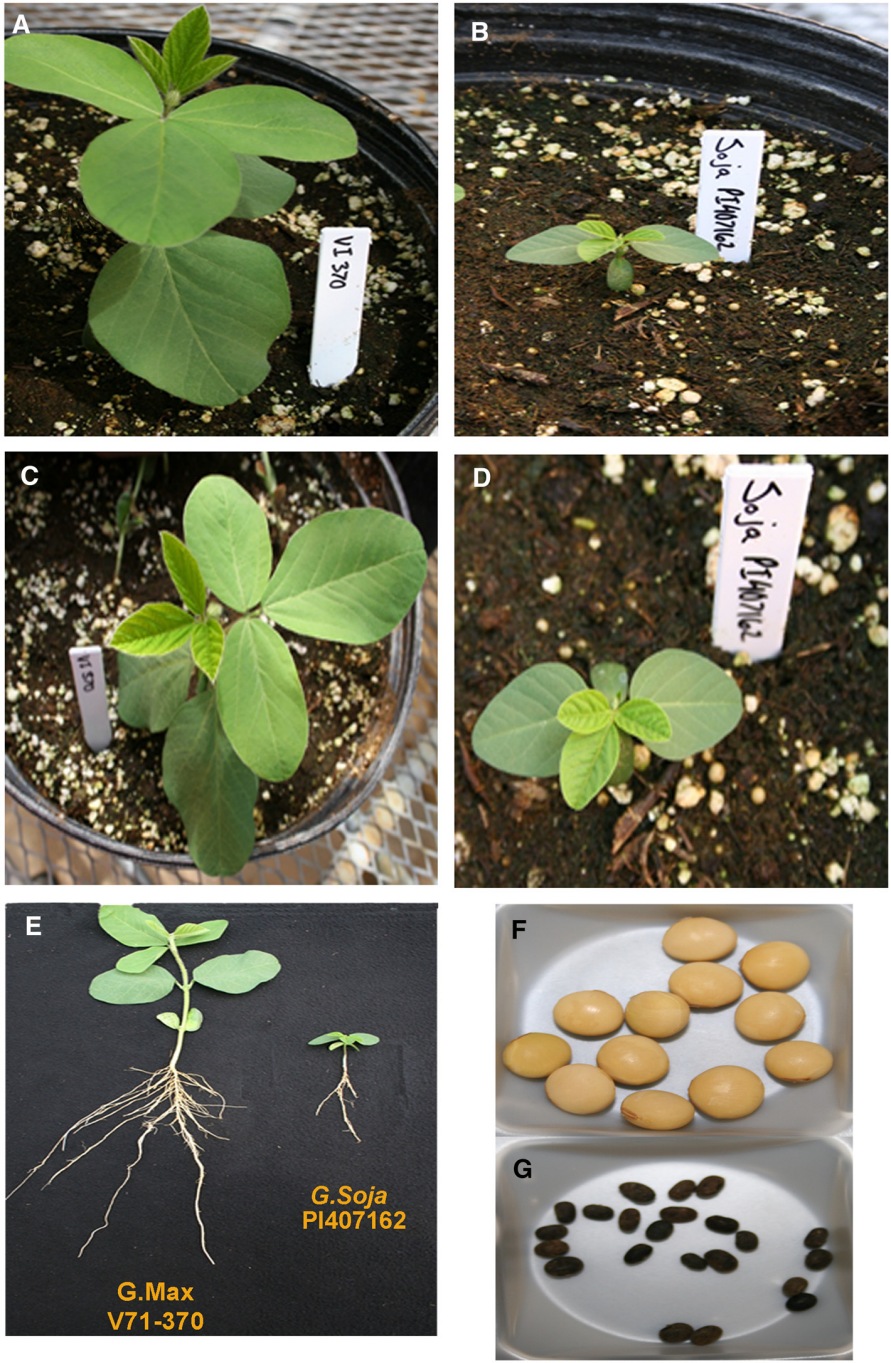
**Variation among parental lines, V71-370 and PI407162 for plant morphology (A-E), first trifoliate leaf size, root architecture, and seed traits (A, C, F: V71-370; B, D, G: PI407162).**

**Figure 2:**
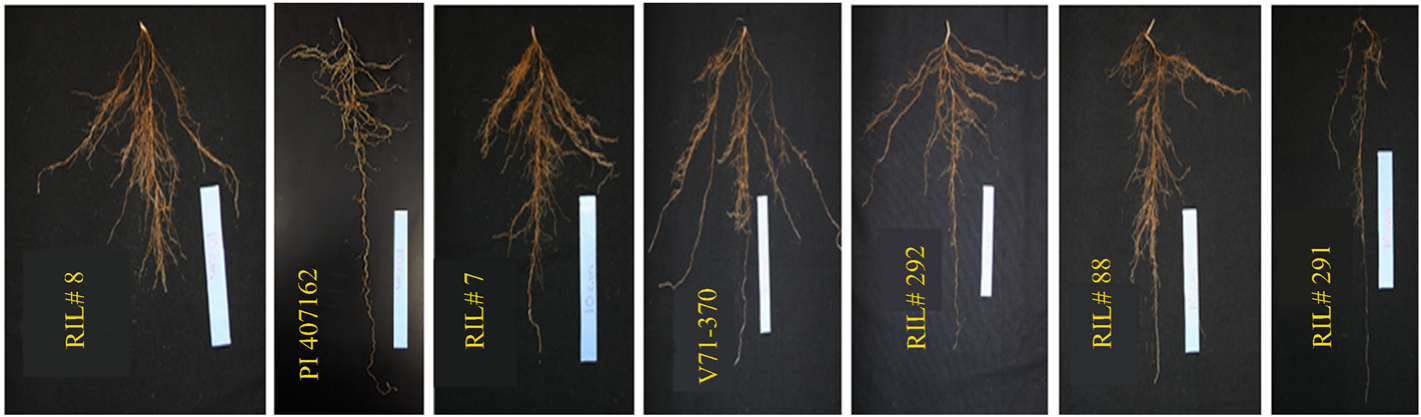
**Transgressive segregation pattern for root traits among recombinant inbred (RI) lines of the mapping population (V71-370/PI407162).**

The parents, V71-370 and PI 407162, show significant variation for a number of common soybean traits including: plant stature, root morphology, and seed size (Figure 1). The *G. max* V71-370 parent develops a larger root system than the wild soybean parent, *G. soja* PI407162. The recombinant inbred lines (RILs) developed from these two parents showed a transgressive segregation for root morphology (Figure 2): many genotypes had longer or shorter taproots and varied total root length compared with the *G. max* and wild parent, respectively (Table 1). The phenotypic mean of all the RILs were similar to the mid-parent values for all remaining traits that were measured. A Shapiro–Wilk test showed that the frequency distributions of these traits were approximately normal (Additional file 1: Figure S1). Most of the previous mapping works in root studies focused on coarse/thicker roots (tap or lateral roots); however, understanding the finer roots and their distribution are important, because they are the ones involved in nutrient and water absorption by increasing the root surface area. Significant positive correlations (P < 0.01) were found amongst various root traits measured in this study (Table 2). The taproot length and tertiary root length were highly correlated (0.8) with root surface area, which influences the plant nutrient and water absorption. Most of the fine roots and their distribution had similar correlations with root volume (Table 2).

**Table 1:** **Phenotypic variation of root traits significant at P value < 0.0001, based on analysis of variance**

**Traits**	**Parental lines**		**RI population**		
	**V71-370**	**PI407162**	**Mean ± SD**	**Minimum**	**Maximum**
Tap root length (cm)	21.5	17.2	24.3 ± 0.42	12.7	36.8
Root fresh weight (g)	2.0	0.4	0.9 ± 0.03	0.2	2.3
Total root length (cm)	425.0	238.0	430.9 ± 11.4	123.4	805.1
Surface area (cm^2^)	73.2	29.1	53.2 ± 1.2	16.3	92.3
Average diameter (mm)	0.6	0.4	0.4 ± 0.01	0.3	0.6
Root volume (cm^3^)	1.0	0.2	0.54 ± 0.01	0.2	1.3
Lateral average diameter (mm)	0.6	0.4	0.5 ± 0.01	0.3	0.7
Tertiary root number	865	439	635.5 ± 22.1	116.0	1650.0
Tertiary root length (cm)	317	191	259.6 ± 8.8	33.5	727.7
Root distribution based on length in diameter (1.0-1.5 mm)	24.0	2.9	9.8 ± 0.4	1.7	28.2
Root distribution based on surface area in diameter (1.0-1.5 mm)	9.2	1.4	3.6 ± 0.2	0.6	10.1
Root distribution based on volume in diameter (1.0-1.5 mm)	0.3	0.04	0.1 ± 0.0	0.0	0.3
Root distribution based on thickness in diameter classification 2 (0.5-1.0 mm)	11.0	0.3	10.8 ± 0.8	0.3	46.5

RI, Recombinant inbred; SD, Standard deviation.

**Table 2:** **Correlation coefficients among various root traits measured in the present study**

**Traits**	**TRTL**	**RFW**	**TRL**	**SA**	**AD**	**RV**	**LAD**	**TERN**	**TERL**	**L3**	**SA3**	**PA3**	**V3**	**T2**
TRTL	1													
RFW	-.199*	1												
TRL	.478**	.005	1											
SA	.420**	.285**	.897**	1										
AD	-.324**	.421**	-.509**	-.147	1									
RV	.239**	.531**	.518**	.832**	.327**	1								
LAD	-.346**	.479**	-.504**	-.138	.792**	.342**	1							
TERN	.132	.152	.741**	.682**	-.338**	.412**	-.314**	1						
TERL	.308**	.176*	.824**	.799**	-.317**	.532**	-.307**	.924**	1					
RDL3	.241**	.389**	.137	.482**	.466**	.739**	.477**	.029	.157	1				
RDSA3	.230**	.399**	.131	.474**	.478**	.738**	.486**	.022	.148	.999**	1			
RDV3	.130	.361**	.100	.413**	.436**	.661**	.457**	.066	.133	.855**	.857**	.853**	1	
RDT2	.421**	-.052	.165*	.220**	.010	.186*	-.083	-.141	.022	.351**	.344**	.340**	.272**	1

Correlations were performed using 160 RI lines of the population. Data used were the means of four replications of independent measurements. Pairwise correlation coefficients were significant at the *5% or **1% significance level.

TRTL, Tap root length (cm); RFW, Root fresh weight (g); TRL, Total root length (cm); SA, Surface area (cm^2^); AD, Average diameter (mm); RV, Root volume (cm^3^); LAD, Lateral average diameter (mm); TERN, Tertiary root number; TERL, Tertiary root length (cm); RDL3, Root distribution in length classification 3 (1.0–1.5 mm); RDSA3, Root distribution in surface area classification 3 (1.0–1.5 mm); RDV3, Root distribution in volume classification 3 (1.0–1.5 mm); RDT2, Root distribution in thickness classification 2 (0.5–1.0 mm).

### Root QTLs and their interaction

**Figure 3:**
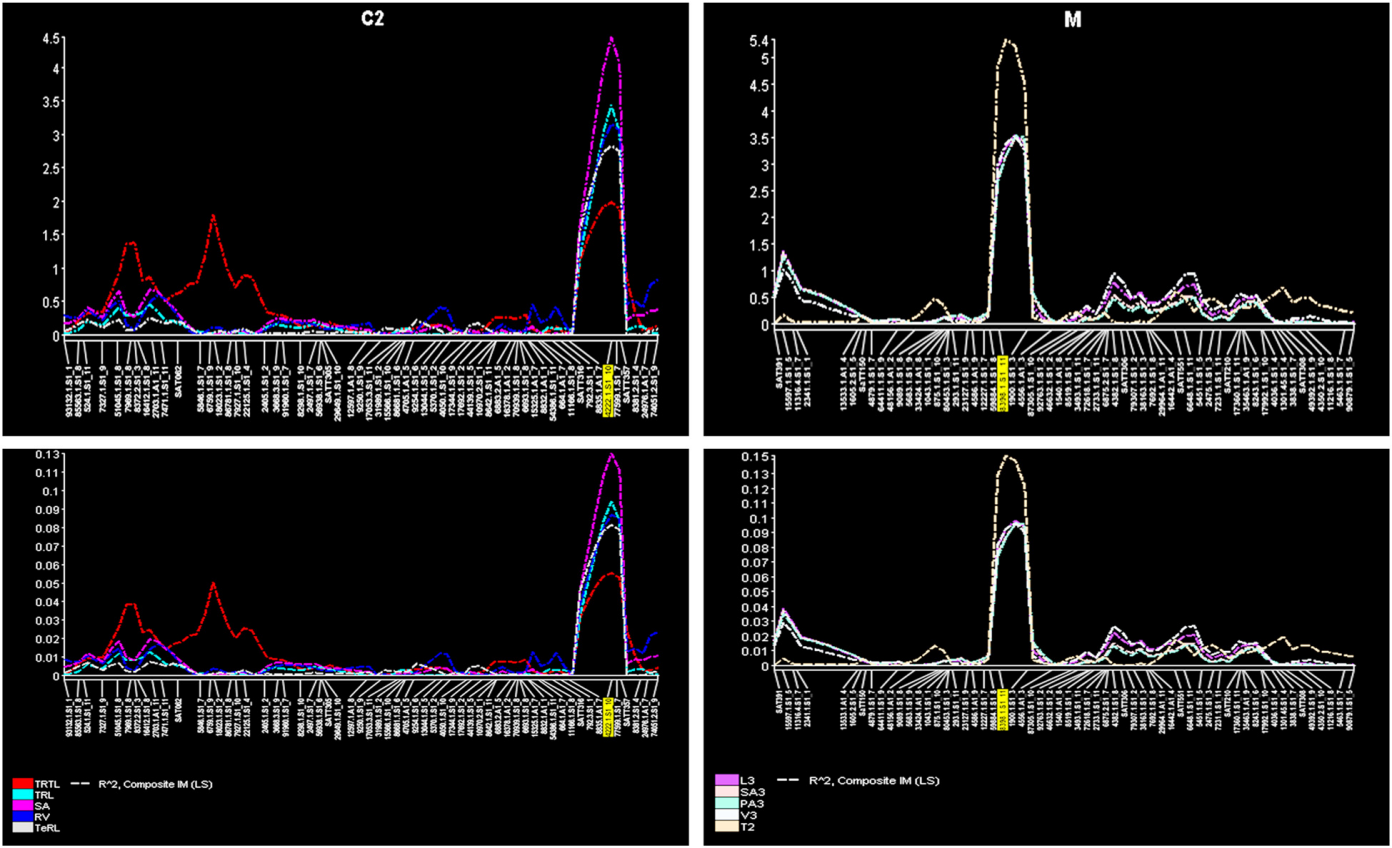
**Significant quantitative trait loci (QTLs) identified on chromosome 6 and 7 for various root architectural traits with their respective R** ^**2**^ **values (panel below).**

**Figure 4:**
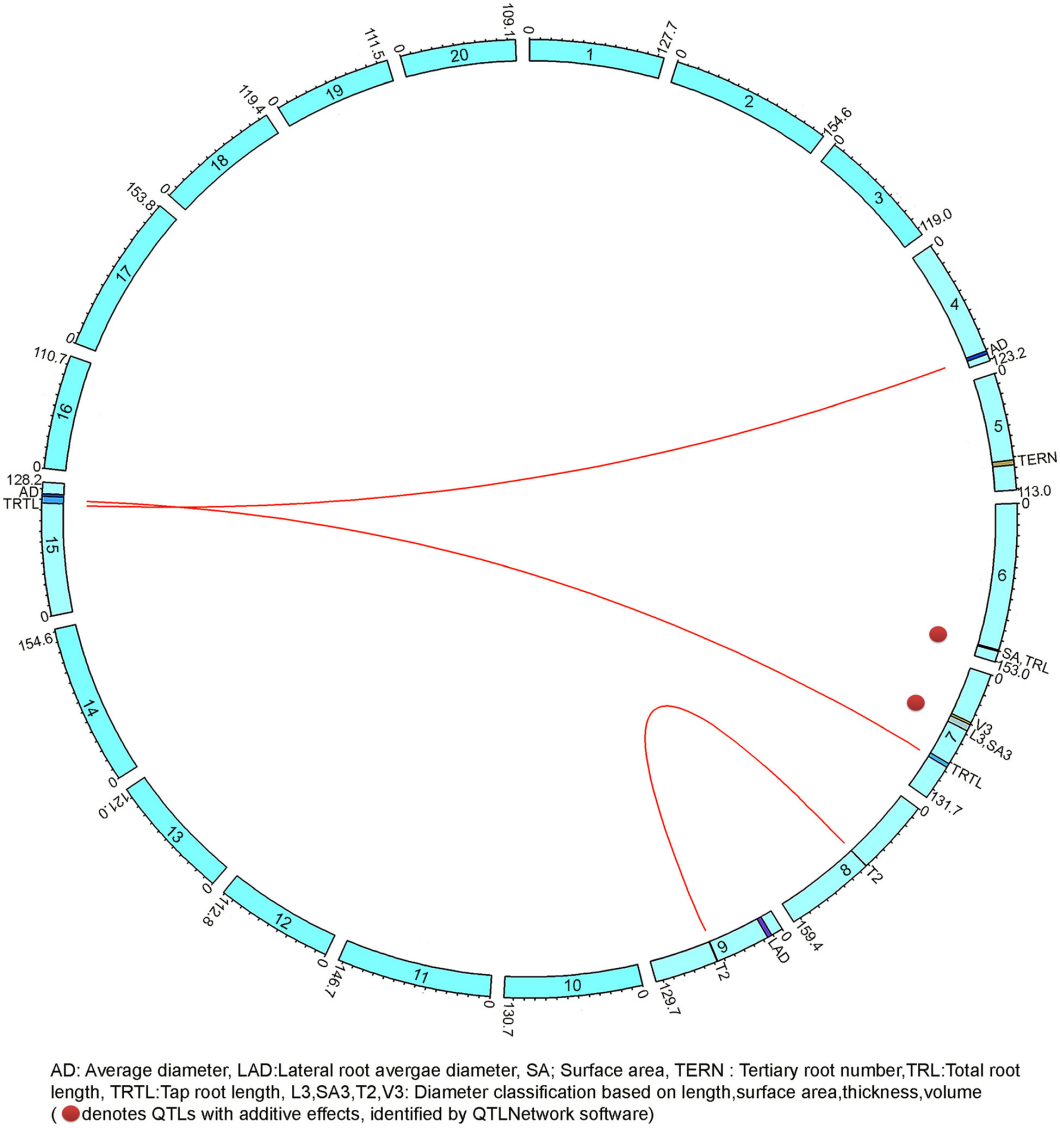
**Circular genome viewer, created using Circos, showing 20 chromosomes with their start and end positions (cM) and denoting different root QTLs and their interactions.**

The root QTLs identified in this study was flanked by Affymetrix probe sets (Table 3). The genes within each QTL interval were selected based on transcript abundance from a previous study [32] and are listed in Table 4. Four significant QTLs were associated with different root architectural traits on chromosomes 6 and 7 (Table 3). Two significant large effect QTLs for root surface area (SA) and thickness (RDT2) were also identified on chromosomes 6 and 7 (Figure 3). The QTL on chromosome 6 (flanked by 4222.1.S1_10 and 77599.1.S1_7) is contributed by PI 407162 and explained >10% of the phenotypic variation for SA and total root length (TRL), with a higher additive effect (Table 3). This array-based mapping approach narrowed the confidence interval of this key QTL region on chromosome 6 to 1.3 cM. This genomic region was also associated with other root traits, such as total root length (Table 3), tertiary root length, and root volume (Additional file 2: Table S1). The root thickness QTL on chromosome 7 (between 59884.1.S1_8 and 8398.1.S1_11) was contributed by V71-370, explaining 15% of the phenotypic variation. The QTL region on chromosome 7 contributed to root distribution in different diameter classes (Table 3) and other traits (Additional file 2: Table S1). Interestingly, both loci had additive effects for their respective traits. Epistatic effects were detected for three pairs of loci (Table 5), and none of these loci were identified as QTLs with single effects. Two loci that contributed to taproot length on chromosome 7 (between 6648.1.S1_11 and 5451.1.S1_5) interacted with the QTL on chromosome 15 (between 6807.1.S1_10 and 9882.1.S1_10) (Figure 4). This epistatic interaction accounted for 8% of the phenotypic variance. A similar interaction was detected for the average diameter between chromosome 4 (between Satt164 and 4792.1.A1_5) and chromosome 15 (between 15910.1.A1_10 and 6807.1.S1_10), which accounted for 7% of the phenotypic variance. For root thickness class 2, an interaction between chromosome 8 (between 70452.1.S1_3 and 55124.1.S1_7) and 9 (between Sat043 and 16443.1.A1_2) was identified that explained 6% of the phenotypic variance.

**Table 3:** **List of large-effect QTLs identified for root architectural traits in the V71-370/PI407162 mapping population using composite interval mapping (CIM) analysis**

**S. No**	**Trait**	**Chr.**	**Marker interval**	**LOD value**	**R** ^**2**^ **value**	**Additive effect**
1	SA	6	4222.1.S1_10 - 77599.1.S1_7	4.5	13.0	−5.23
2	TRL	6	4222.1.S1_10 - 77599.1.S1_7	3.5	11.0	−46.2
3	RDT2	7	59884.1.S1_8- 8398.1.S1_11	5.1	15.0	3.40
4	RDL3	7	8398.1.S1_11 - 1900.1.S1_3	3.6	10.0	1.71

SA, Surface area (cm^2^); TRL, Total root length (cm); RDT2, Root distribution in thickness classification 2 (0.5–1.0 mm); RDL3, Root distribution in length classification 3 (1.0–1.5 mm).

**Table 4:** **List of genes identified within each potential root QTL interval based on transcript abundance in microarray analyses**

**S. no**	**Affymetrix probe ID**	Annotation based on expression profiling*	**Gene ID**	**Gene details #**	**Root QTLs flanked**
1	Gma.4222.1.S1_10	Probable carboxylesterase 6-like	*Glyma06g46680*	Alpha/beta hydrolase	Surface area, Total length
2	GmaAffx.77599.1.S1_7	Uncharacterized	*Glyma06g46850*	Histone-like CCAAT Transcription Factor	
3	GmaAffx.59884.1.S1_8	Kinesin-like protein KIF2C-like	*Glyma07g09530*	Kinesin like protein	Root length (1.0–1.5 mm)
			*Glyma09g32280*		Root thickness (0.5–1.0 mm)
4	Gma.8398.1.S1_11	Lipase 1-like	*Glyma07g09860*	Triglyceride lipase-cholesterol esterase	
			*Glyma09g31950*		
5	Gma.1900.1.S1_3	DEAD-box ATP-dependent RNA helicase 20-like	*Glyma07g11880*	ATP-dependent RNA helicase	
			*Glyma08g20670*		
			*Glyma07g01260*		
6	Gma.6648.1.S1_11	Apoptosis inhibitor 5-like	*Glyma07g32480*	Apoptosis Inhibitor 5-related	Tap root length
			*Glyma13g24090*		
7	Gma.5451.1.S1_5	Uncharacterized	*Glyma02g15190*	Apoptosis-promoting RNA-binding protein	
			*Glyma07g33300*		
8	Gma.6807.1.S1_10	Cysteine synthase-like	*Glyma15g41600*	Cystathionine beta-synthase and related enzymes	
9	Gma.9882.1.S1_10	Uncharacterized	*Glyma15g42220*	slow anion channel associated 1-like	
10	Gma.4792.2.S1_5	Uncharacterized	*Glyma04g42630*	BTB domain transcription factor	Average diameter
			*Glyma06g12140*		
11	Gma.15910.1.S1_10	Uncharacterized	*Glyma11g26970*	Nuclear distribution protein NUDC	
			*Glyma18g07050*		
	Gma.6807.1.S1_10	Cysteine synthase-like	*Glyma15g41600*	Cystathionine beta-synthase and related enzymes	
12	GmaAffx.70452.1.S1_3	Calmodulin-binding transcription activator 2-like	*Glyma08g19100*	CAMTA Transcription factor	Root thickness (0.5–1.0 mm)
			*Glyma15g05900*		
13	GmaAffx.55124.1.S1_7	Metacaspase-1	*Glyma08g19050*	Metacaspase involved in regulation of apoptosis	
14	Gma.16443.1.A1_2	Histone-lysine N-methyltransferase ASHH3-like	*Glyma09g28430*	Uncharacterized	

*The full expression profiling data of mock control plants can be accessed from the NCBI database. (http://www.ncbi.nlm.nih.gov/geo/query/acc.cgi?acc=GSE11611).

# The gene annotation information was from SoyKB.

**Table 5:** **Estimated additive × additive epistatic effect QTLs detected by QTLNetwork for root architectural traits**

**Trait**	**Chr.**	**Marker interval**	**Chr.**	**Marker interval**	**Epistasis**	**h** ^**2**^ **(aa)**
TRTL	7	6648.1.S1_11 -5451.1.S1_5	15	6807.1.S1_10 – 9882.1.S1_10	−1.52	0.08
AD	4	Satt164-4792.1.A1_5	15	15910.1.A1_10 - 6807.1.S1_10	0.02	0.07
RDT2	8	70452.1.S1_3 – 55124.1.S1_7	9	Sat043 – 16443.1.A1_2	2.41	0.06

TRTL, Tap root length (cm); AD, Average diameter (mm); RDT2, Root distribution in thickness classification 2 (0.5–1.0 mm).

### Identification of genes associated with the root QTLs

**Figure 5:**
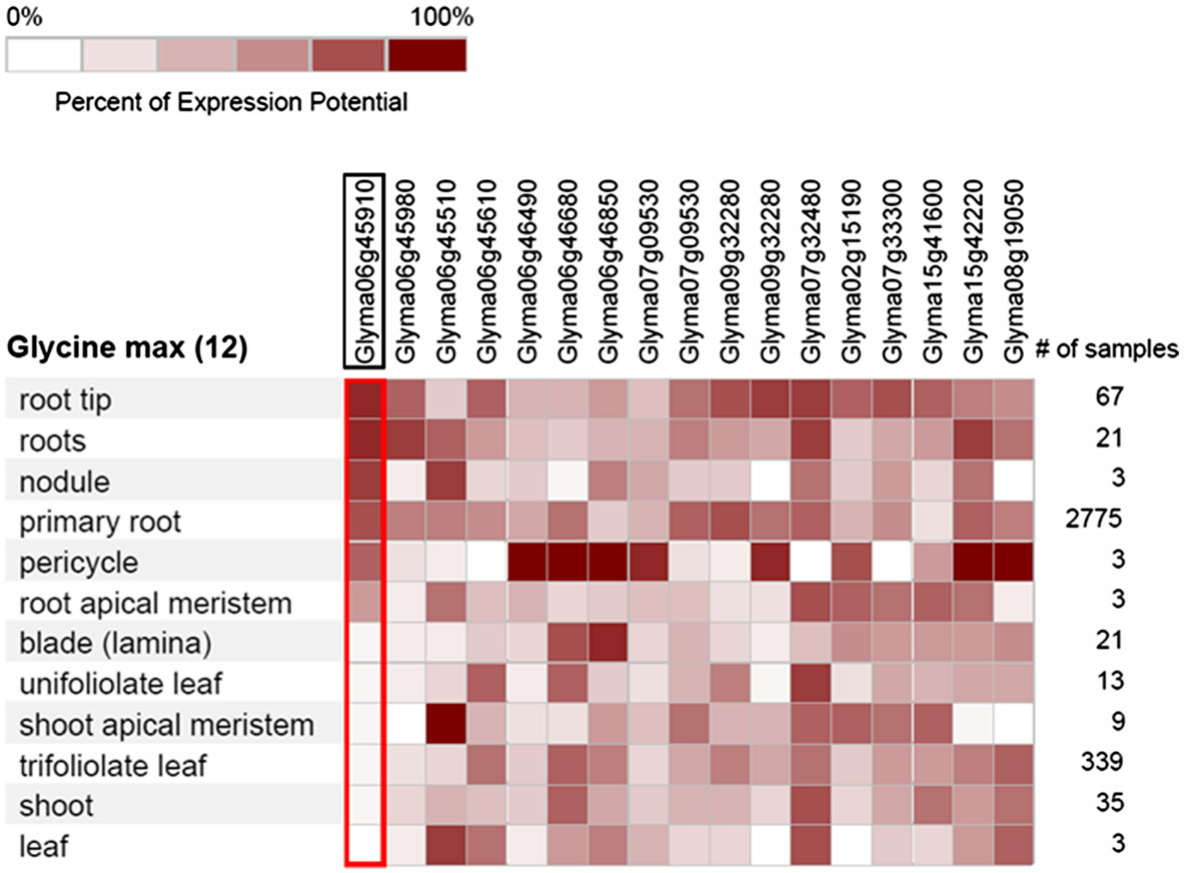
**Heat map of all genes identified in this study and their gene chip expression pattern in 12 different soybean tissues, derived using the Genevestigator software.**

Twenty-three genes were selected based on their transcript abundance (Table 4) in the microarray analysis [32] within the mapped QTL intervals (Tables 3 and 5; Additional file 2: Table S1). To study the genes specific to the wild soybean within the QTL interval on chromosome 6, sequence information of 162 genes (Table 6) were extracted from another *G. soja* accession, IT182932 [8] and annotated as shown in Additional file 3: Figure S2. The sequence of each of the 162 genes was compared between Williams 82 with another *G. soja* accession; 18 of these genes (Tables 7 and 8) were selected for expression analysis using qRT-PCR. Nine of the 18 genes (Table 7) had high transcript abundance in root tissues already in the soybean transcriptome database [33], while the remaining nine genes had non-synonymous mutations (Table 8). Based on the normalized tissue-specific expression pattern in root tissues in the soybean Affymetrix gene chip dataset [34], key genes were identified (Figure 5) for quantitative RT-PCR analysis.

**Table 6:** **Genes selected from wild soybean variety IT182932** ^**a**^ **, based on the QTL confidence interval on chromosome 6, with their annotations**

**Gene ID**	**Annotation**
*Glyma06g44810*	Tetraspanin family protein
*Glyma06g44980*	Epoxide hydrolase 2-like
*Glyma06g44010*	8-hydroxyquercetin 8-o-methyltransferase-like isoform 1
*Glyma06g44660*	Transcription factor bhlh36-like
*Glyma06g44990*	Epoxide hydrolase 2-like
*Glyma06g44600*	O-acyltransferase wsd1
*Glyma06g44650*	Alpha-farnesene synthase
*Glyma06g44830*	Accelerated cell death 6
*Glyma06g44890*	Protein
*Glyma06g44800*	Ribosomal-protein-alanine acetyltransferase-like
*Glyma06g44620*	ATP synthase mitochondrial f1 complex assembly factor 1-like
*Glyma06g44770*	MYB Transcription factor
*Glyma06g44780*	Hypothetical protein PRUPE_ppa014299mg
*Glyma06g44970*	Gdsl esterase lipase exl3-like
*Glyma06g44930*	Expansin-b3-like precursor
*Glyma06g44730*	Probable serine threonine-protein kinase at1g54610-like
*Glyma06g44740*	PREDICTED: uncharacterized protein LOC100805467
*Glyma06g44630*	Mitochondrial dihydroorotase
*Glyma06g44880*	Ankyrin repeat-containing protein at3g12360-like
*Glyma06g44790*	Caax amino terminal protease family protein
*Glyma06g44720*	L-type lectin-domain containing receptor kinase -like
*Glyma06g44750*	Mannan endo-beta-mannosidase 2-like
*Glyma06g44640*	O-acyltransferase wsd1-like
*Glyma06g44900*	Ankyrin repeat-containing protein at3g12360-like
*Glyma06g45100*	Probable protein phosphatase 2c 52-like
*Glyma06g45280*	Wound-induced protein
*Glyma06g45210*	Micronuclear linker histone
*Glyma06g45590*	G-type lectin s-receptor-like serine threonine-protein kinase
*Glyma06g45560*	Myb-related protein myb4-like
*Glyma06g45910*	Peroxidase 3
*Glyma06g45820*	Riboflavin synthase alpha chain
*Glyma06g45860*	Xyloglucan endotransglucosylase hydrolase protein 9
*Glyma06g45740*	Probable histone-lysine n-methyltransferase atxr3-like
*Glyma06g45300*	Unnamed protein product
*Glyma06g45420*	Wound-induced protein
*Glyma06g45230*	Tpa: duf566 domain containing family protein
*Glyma06g45720*	Formin-like protein 5-like
*Glyma06g45430*	Wound-induced protein
*Glyma06g45290*	Uncharacterized loc101222779
*Glyma06g45920*	Peroxidase 3
*Glyma06g45800*	Proline-rich protein
*Glyma06g45550*	Myb-related protein myb4-like
*Glyma06g45130*	Protein
*Glyma06g45390*	Wound-induced protein
*Glyma06g45350*	Wound-responsive protein
*Glyma06g45650*	F-box family protein
*Glyma06g45090*	Low quality protein: uncharacterized loc101222318
*Glyma06g45770*	Btb poz domain-containing protein at3g22104-like
*Glyma06g45410*	Wound-induced protein
*Glyma06g45980*	Uncharacterized protein LOC100786184 (Predicted)
*Glyma06g45020*	Uncharacterized protein LOC100305963
*Glyma06g45310*	Embryo defective 1923 protein
*Glyma06g45000*	Probable polyol transporter 4-like
*Glyma06g45260*	Uncharacterized loc101222779
*Glyma06g45150*	Protein strubbelig-receptor family 3-like
*Glyma06g45670*	Hypothetical protein MTR_052s0005
*Glyma06g45450*	Diacylglycerol kinase 5
*Glyma06g45830*	Uncharacterized loc101209217
*Glyma06g45850*	E3 ubiquitin-protein ligase rma1h1-like
*Glyma06g45370*	Wound-induced protein
*Glyma06g45520*	Myb-related protein myb4-like
*Glyma06g45840*	Gpi-anchored protein
*Glyma06g45380*	Wound-induced protein
*Glyma06g45960*	Plant cell wall protein 88
*Glyma06g45680*	Dehydration responsive element binding protein
*Glyma06g45620*	Zinc finger protein constans-like protein
*Glyma06g45640*	Indole-3-acetic acid-amido synthetase -like
*Glyma06g45810*	Casp-like protein rcom_1174750-like
*Glyma06g45730*	Uncharacterized loc101212188
*Glyma06g45220*	Uncharacterized protein LOC100527304
*Glyma06g45400*	Uncharacterized loc101222779
*Glyma06g45050*	Caffeic acid 3-o-methyltransferase
*Glyma06g45010*	Drought responsive element binding protein 5
*Glyma06g45120*	Probable indole-3-acetic acid-amido synthetase -like
*Glyma06g45160*	Secretory carrier-associated membrane protein 1-like
*Glyma06g45700*	Beta-amylase
*Glyma06g45440*	Protein thylakoid chloroplastic-like
*Glyma06g45110*	Cell wall
*Glyma06g45990*	Ring-box protein 1a-like
*Glyma06g45490*	Equilibrative nucleoside transporter
*Glyma06g45890*	Trab domain-containing
*Glyma06g45140*	Unknown function
*Glyma06g45880*	Transferring glycosyl
*Glyma06g45930*	Translation initiation factor
*Glyma06g45170*	H aca ribonucleoprotein complex non-core subunit naf1
*Glyma06g45940*	Triptychon and
*Glyma06g45510*	D6-type cyclin
*Glyma06g45950*	Isocitrate lyase
*Glyma06g45480*	Uncharacterized gpi-anchored protein at4g28100-like
*Glyma06g45780*	Isoprene synthase
*Glyma06g45360*	Vesicle-associated protein 4-2-like
*Glyma06g45200*	Xylosyltransferase 1-like
*Glyma06g45570*	Myb-related protein myb4-like
*Glyma06g45240*	Wound-induced protein
*Glyma06g45460*	Myb-related transcription factor
*Glyma06g45710*	Pentatricopeptide repeat-containing protein at1g08070-like
*Glyma06g45080*	Elmo domain-containing protein a-like
*Glyma06g45610*	Outer arm dynein light chain 1 protein
*Glyma06g45540*	Myb-related protein myb4-like
*Glyma06g45580*	Uncharacterized protein LOC100781575 (Predicted)
*Glyma06g45340*	Nad h dehydrogenase mitochondrial-like
*Glyma06g45180*	Protein ultrapetala 1-like
*Glyma06g46540*	Replication factor c subunit 1-like
*Glyma06g46500*	Uncharacterized protein LOC100796231 (Predicted)
*Glyma06g46020*	Duf246 domain-containing protein
*Glyma06g46550*	Adipocyte plasma membrane-associated
*Glyma06g46530*	Fasciclin-like arabinogalactan protein 17-like
*Glyma06g46350*	L-ascorbate oxidase homolog
*Glyma06g46370*	B-cell receptor-associated 31-like protein
*Glyma06g46880*	Pentatricopeptide repeat-containing protein at1g11290-like
*Glyma06g46360*	Unknown function
*Glyma06g46450*	Cellulose synthase-like protein h1
*Glyma06g46960*	Uncharacterized protein LOC100814328 (Predicted)
*Glyma06g46520*	Probable carboxylesterase 15-like
*Glyma06g46660*	Rj2 protein
*Glyma06g46490*	Outer envelope protein chloroplastic-like
*Glyma06g46650*	Protein
*Glyma06g46600*	Choline ethanolamine kinase
*Glyma06g46150*	Protein transparent testa 12-like
*Glyma06g46590*	Myb transcription factor myb142
*Glyma06g46110*	Upf0481 protein at3g47200-like
*Glyma06g46340*	Mip sip subfamily
*Glyma06g46320*	Zinc finger ccch domain-containing protein 13-like
*Glyma06g46290*	Protein
*Glyma06g46740*	Auxin-induced protein 5 ng4-like
*Glyma06g46710*	Sister chromatid cohesion protein dcc1-like
*Glyma06g46640*	Transcription initiation factor tfiid subunit 7-like
*Glyma06g46750*	Cytochrome p450
*Glyma06g46190*	Aconitate cytoplasmic-like
*Glyma06g46610*	Ring-h2 finger protein atl69-like
*Glyma06g46270*	Autophagy-related protein 8c-like
*Glyma06g46120*	Septum-promoting gtp-binding protein 1-like
*Glyma06g46400*	S-type anion channel slah1-like
*Glyma06g46160*	Uncharacterized gpi-anchored protein at1g61900-like
*Glyma06g46210*	Nedd8-activating enzyme e1 regulatory subunit-like
*Glyma06g46260*	Upf0481 protein at3g47200-like
*Glyma06g46620*	Ribosomal l5e family protein
*Glyma06g46680*	Probable carboxylesterase 6-like
*Glyma06g46220*	Rrp6-like protein 3
*Glyma06g46380*	Disease resistance response protein 206-like
*Glyma06g46580*	Uncharacterized protein LOC100527051
*Glyma06g46180*	Succinate dehydrogenase subunit 3
*Glyma06g46430*	Protein usf-like
*Glyma06g46630*	Protein
*Glyma06g46390*	Disease resistance response protein 206-like
*Glyma06g46760*	Cytochrome p450
*Glyma06g46300*	Zinc metalloprotease slr1821-like
*Glyma06g46240*	Upf0481 protein at3g47200-like
*Glyma06g46410*	Mitogen-activated protein kinase kinase kinase a-like
*Glyma06g46470*	At1g05070 t7a14_6
*Glyma06g46090*	Upf0481 protein at3g47200-like
*Glyma06g46250*	Septum-promoting gtp-binding protein 1-like
*Glyma06g46170*	Uncharacterized protein LOC100779566 (Predicted)
*Glyma06g46480*	Low quality protein: condensin complex subunit 2-like
*Glyma06g46130*	Glutamate receptor -like
*Glyma06g46570*	Proline-rich family protein
*Glyma06g46730*	Ring-h2 finger protein atl51-like
*Glyma06g46420*	WRKY transcription factor
*Glyma06g46000*	Homeobox-leucine zipper protein hdg2-like
*Glyma06g46230*	Probable beta- -galactosyltransferase 2-like
*Glyma06g46560*	Yabby2-like transcription factor yab2
*Glyma06g46690*	Signal peptidase complex catalytic subunit sec11c-like

^a^The genes specific to *G. soja with a* deleterious mutation were obtained from Kim et al. [6,8].

**Table 7:** **List of genes with transcript abundance in root tissues alone selected on chromosome 6 QTL intervals, based on re-sequence data on** ***G. soja*** **variety IT182932** ^**a**^

**Gene ID**	**Gene annotation** ^**a**^	Genome status*
*Glyma06g44981*	Epoxide hydrolase 2-like	N/A
*Glyma06g44880*	Ankyrin repeat-containing protein	Duplicated
*Glyma06g44900*	Ankyrin repeat-containing protein	Duplicated
*Glyma06g45910*	Peroxidase 3	Duplicated
*Glyma06g45920*	Peroxidase 3	Single copy
*Glyma06g45980*	Uncharacterized protein	N/A
*Glyma06g45261*	Uncharacterized protein	N/A
*Glyma06g45810*	CASP like protein	Single copy
*Glyma06g46170*	Uncharacterized protein	Duplicated

*Information on genome duplication and copy number variation was obtained from Du et al. [56].

^a^The genes specific to *G. soja* were obtained from Kim et al. [6,8].

**Table 8:** **List of genes with a deleterious mutation selected on chromosome 6 QTL intervals based on re-sequence data on** ***G. soja*** **variety IT182932** ^**a**^

**Gene ID**	**Gene annotation** ^**a**^	Genome status*
*Glyma06g45510*	G1/S-specific cyclin D	Duplicated
*Glyma06g45610*	Leucine rich repeat	Duplicated
*Glyma06g45740*	Histone H3 (Lys4) methyltransferase complex	Duplicated
*Glyma06g45850*	RING finger	Single copy
*Glyma06g45890*	TraB family proteins	Duplicated
*Glyma06g46210*	NEDD8-activating complex	Duplicated
*Glyma06g46490*	TPR Transcription factor	Duplicated
*Glyma06g46710*	Unknown function	Single copy
*Glyma06g46730*	Zinc finger, C3HC4 type (RING finger)	Duplicated

*Information on genome duplication and copy number variation was obtained from Du et al. [56].

^a^The genes specific to *G. soja* with a deleterious mutation were obtained from Kim et al. [6,8].

### Presence of non-synonymous mutation in root-related genes

Sequence data for the parental lines V71-370 and PI 407162 were analyzed for the presence of non-synonymous SNPs within the 23 selected genes, based on transcript abundance from a microarray analysis, to identify candidate genes that might contribute to variation in root phenotypes. Only three of these genes (Table 4), had non-synonymous SNPs, two in the *G. soja* line, PI 407162 (*Glyma07g09860 and Glyma15g42220*) and one gene (*Glyma07g32480*) in both parental lines that had altered the amino acid content (Table 9). The gene *Glyma07g09860* encodes triglyceride lipase, showed higher transcript abundance and was in the QTLs identified for root distribution based on length and thickness (Table 3). The remaining two genes, *Glyma07g32480* and *Glyma15g42220*, encode apoptosis inhibitory 5 family protein and oxidoreductase/transition metal ion binding protein, respectively. These two genes also showed higher transcript abundance and mapped to the taproot length QTL intervals. Missense mutations were also identified in four of 18 genes (Table 8) with significant differences in transcript abundance selected within the root total length and surface area QTL confidence interval on chromosome 6 (Table 7) (*Glyma06g45920, Glyma06g44900, Glyma06g46170, and Glyma06g45910*) (Table 9). There was an insertion/deletion variation (Indel) in the coding sequence of two genes from the *G. soja* parent, *Glyma06g45510* (insertion) and *Glyma06g45261* (deletions), which were in the cell cycle-associated *D6 type cyclin* gene and the key hormone auxin-associated gene, *auxin efflux carrier protein* gene. Five out of nine genes (Table 8) selected from another wild soybean IT182932 [8] had similar conserved missense mutations (Table 10) to those in the wild soybean, PI407162, used in this study. *Glyma06g46490* encodes a TPR transcription factor with high expression that is limited to the root pericycle cells (Figure 5).

**Table 9:** **List of genes associated with root traits and with the non-synonymous SNPs in V71-370 and PI 407162 parental lines**

**S. No**	Gene ID and annotation*	**SNP position**	**W82 (Ref. genome)**	**V71-370**	**PI407162**	**AA change**
1	*Glyma07g09860* ^*+*^ Triglyceride lipase-cholesterol esterase	8318354	A	A	C	Phenylalanine to Cysteine
2	*Glyma07g32480* ^*+*^ Apoptosis Inhibitor 5-related	37386211	G	T	G	Proline to Threonine
		37386231	C	T	C	Glycine to Glutamic Acid
		37388170	C	C	G	Glutamic Acid to Glutamine
3	*Glyma15g42220* ^*+*^ Uncharacterized	49621619	C	C	T	Glutamic Acid to Lysine
		49621846	C	C	T	Arginine to Glutamine
4	*Glyma06g45920* Peroxidase 3	48646118	C	C	A	Arginine to Leucine
5	*Glyma06g44900* Ankyrin repeat-containing protein	47715303	A	G	A	Glutamic Acid to Glycine
6	*Glyma06g46170* Uncharacterized	48839708	G	G	C	Glycine to Alanine
		48839816	G	G	A	Serine to Asparagine
		48839842	G	G	A	Glutamic Acid to Lysine
		48840059	G	G	A	Tryptophan (stop gained in wild soybean)
7	*Glyma06g45910* Peroxidase 3	48635068	A	A	C	Serine to Alanine
8	*Glyma06g46210* NEDD8-activating complex	48869473	A	A	C	Lysine to Asparagine
		48873032	C	C	G	Arginine to Cysteine
		48873041	C	C	A	Alanine to Glycine
		48874136	A	A	G	Threonine to Asparagine
9	*Glyma06g45261* Uncharacterized	48049483	A	G	A	Histidine to Arginine

*Genes selected based on Affymetrix probe hybridization data are denoted with a + sign; the other genes were selected based on QTL confidence intervals.

**Table 10:** **Conserved non-synonymous mutations in root-related genes among wild soybean varieties**

**S. No**	Gene ID/Annotation*	**SNP position**	**W82 (Ref. genome)**	**V71-370**	**PI407162**	**AA change**	**Similar non-Synonymous SNPs in other wild soybeans** ^**+**^
1	*Glyma06g46210* NEDD8-activating complex	48869473	A	A	C	Lysine to Asparagine	W09
		48872310	C	C	T	Arginine to Cysteine	W05, W06, W08, W12, W13, W14, W16
		48873032	C	C	G	Alanine to Glycine	W05, W08, W09, W12, W13, W14, W16, W17
		48873041	C	C	A	Threonine to Asparagine	W05, W08, W09, W12, W13, W14, W16
		48874136	A	A	G	Lysine to Arginine	W05, W06, W08, W09, W12, W13, W14, W16,W17
2	*Glyma06g45510* G1/S-specific cyclin D	48234941	G	G	A	Aspartic Acid to Asparagine	All lines except W12
		48235772	A	G	A	Methionine to Valine	W01, W10, W12, W17
		48236876	C	C	A	Histidine to Asparagine	W01, W02, W03, W04, W05, W07, W08, W09, W11, W13, W15, W16, W17
3	*Glyma06g45610* Leucine rich repeat	48346924	C	T	C	Glycine to Aspartic acid	W01, W02, W03, W04, W05, W06, W07, W08, W12, W13, W15, W16, W17
		48353134	G	G	T	Threonine to Asparagine	W05, W09, W12, W11, W16
4	*Glyma06g46490* TPR Transcription factor	49095540	G	A	G	Aspartic Acid to Asparagine	All except W07, W09 and W10
		49095620	T	A	T	Serine to Arginine	W10
		49095627	A	G	A	Asparagine to Aspartic Acid	W01, W03, W06, W08, W09
		49095824	A	G	A	Threonine to Alanine	All except W10, W13, W17
		49096075	A	G	A	Isoleucine to Methionine	All except W10
5	*Glyma06g46730* C3HC4 type (RING finger)	49310698	T	T	C	Phenylalanine to Serine	W10, W13, W14, W16
		49310719	C	C	T	Alanine to Valine	W09, W10, W12, W13, W14, W16, W17
		49310842	G	G	A	Arginine to Glutamine	All except W07, W10, W09, W13, W14, W17
		49311027	A	A	G	Isoleucine to Valine	All except W07, W09, W10, W12, W13, W14, W17
		49311204	C	C	G	Glutamine to Glutamic Acid	W12, W13, W14, W16, W17

^+^The wild soybean designations were derived from Lam et al. [35].

### Expression patterns of root-related genes

**Figure 6:**
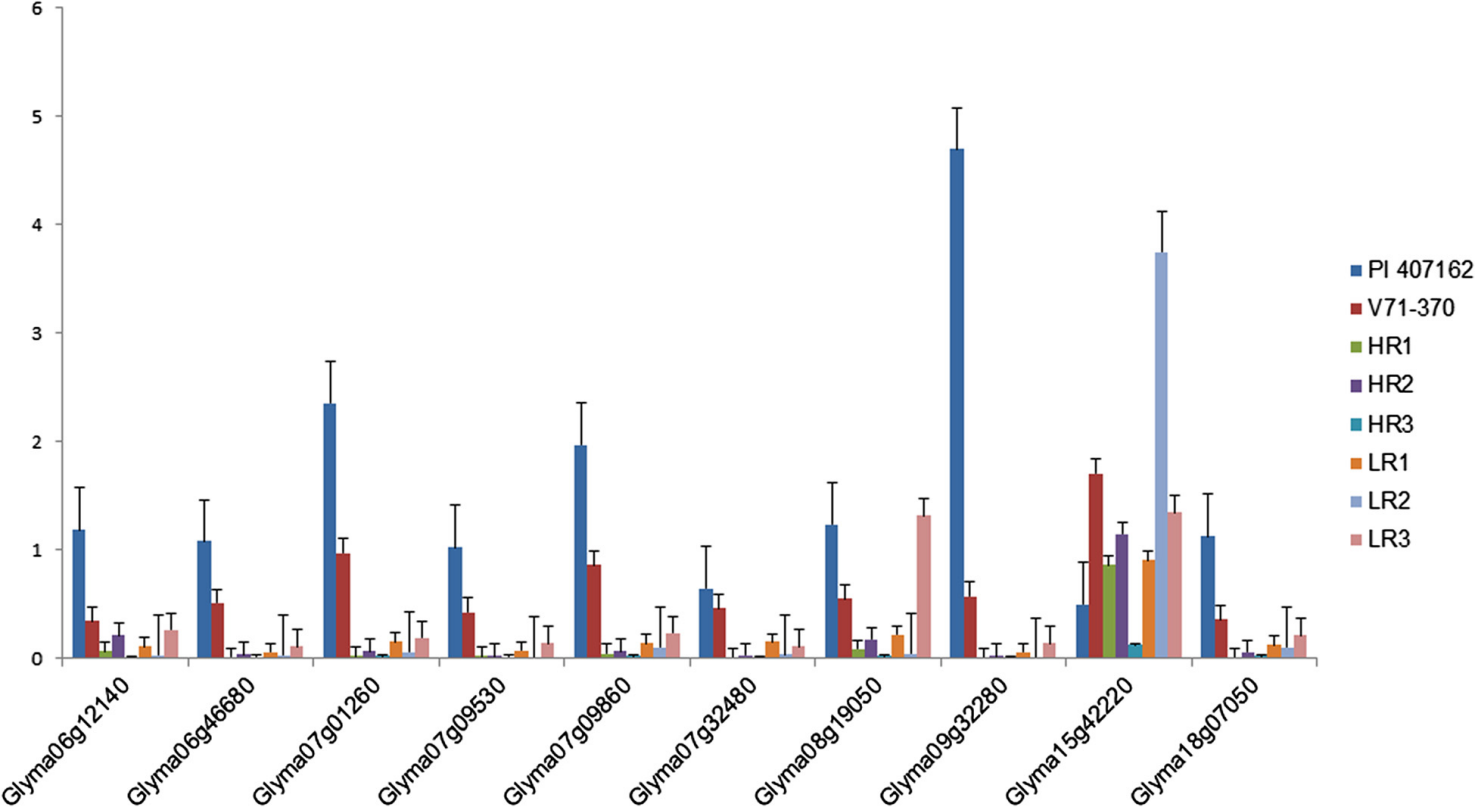
**Expression patterns of genes with high transcript abundance within the root QTL peaks detected among parental lines and selected Recombinant Inbred Lines (RILs) selected.**
  Eighteen genes within the QTL region on chromosome 6 were selected based on sequence polymorphisms with *G. soja* accession IT182932 (Tables 7 and 8). Among nine genes listed in Table 7, three *Glyma06g45910, Glyma06g45980*, and *Glyma06g44880*, showed higher expression in the *G. soja* parent PI 407162 (Figure 7A); and four genes, *Glyma06g44900, Glyma06g45261, Glyma06g45810*, and *Glyma06g45920*, showed higher expression in the *G. max* parent V71-370 (Figure 7A). Interestingly, an uncharacterized protein (*Glyma06g45980*) gene had higher expression levels in the *G. soja* parent than in *G. max*. The remaining nine genes in the QTL region, each with a non-synonymous mutation, (Table 8) showed higher expression in *G. max* than in *G. soja*, except for *Glyma06g46210* (Figure 8). Based on 15× depth sequence data from PI 407162, five genes exhibited missense mutations (Table 10). These mutations were conserved when this sequence was compared with other public sequence databases of diverse *G. soja* lines from Korea [8] and China [35].

- ***Parental genotypes***
  The differential expression of root-related genes from the *G. soja* accession and cultivated soybean parent enabled us to gain an understanding of the gene regulation associated with various root architecture traits. Based on transcript abundance, 10 genes were identified with significant expression fold changes among the parental lines (Figure 6). Nine genes had a significantly higher expression in the *G. soja* parental line compared with the *G. max* parent, while gene *Glyma15g42220* showed the opposite trend. When sequence comparisons were made, three genes (*Glyma07g09860, Glyma07g32480*, and *Glyma15g42220*) had non-synonymous SNPs (Table 9). Only the gene *Glyma07g32480* had a non-synonymous mutation in both parental lines. The *kinesin motor family protein* (*Glyma09g32280*) had the highest expression in wild soybean, which may have contributed to the smaller and finer roots.

**Figure 7:**
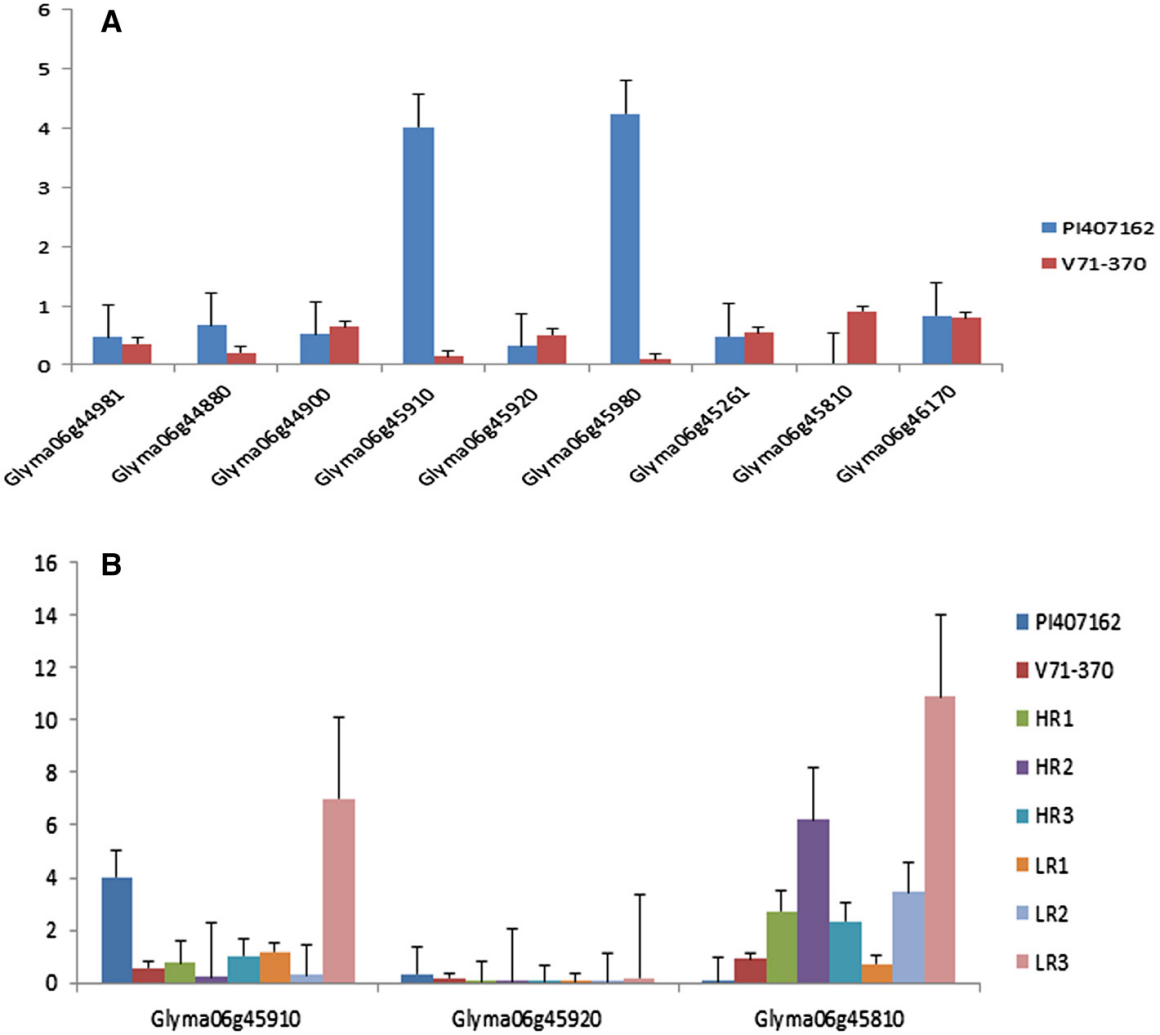
**Expression pattern of root specific candidate genes on chromosome 6. (A)** Parental lines expression **(B)** Expression of two Peroxidase and Casp like protein genes.

**Figure 8:**
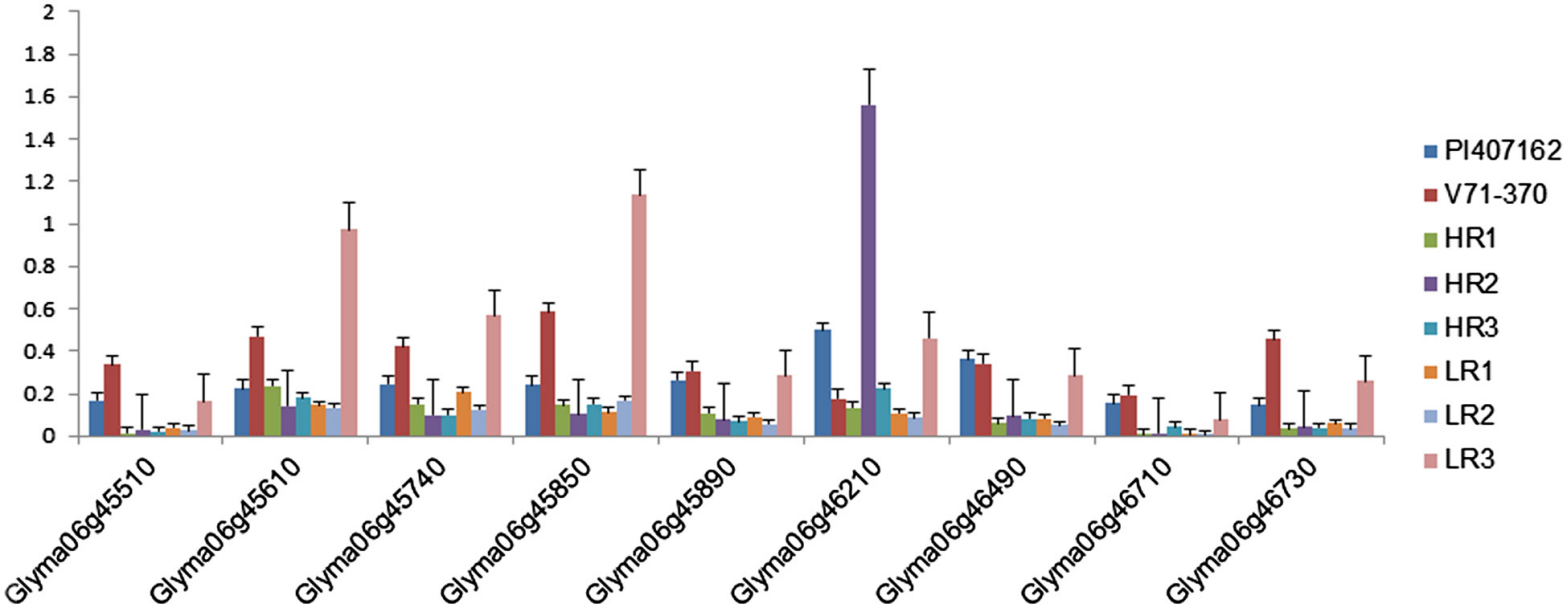
**Expression patterns of genes with a deleterious mutation on chromosome 6 among parental lines and selected RILs.**
- ***RILs with extreme root phenotypes***
  Eleven genes were selected based on transcript abundance (Table 4) from within the QTL intervals on chromosome 7 for the root diameter distribution based on length and thickness (Table 3) Three of these genes, *Kinesin like proteins*, *triglyceride lipase*, and *ATP-dependent RNA helicase* showed higher expression in RILs with the smallest root phenotypes. These three genes could represent prime candidate genes that play a critical role in regulating fine root development and distribution based on length and thickness. The additive effect taproot length QTL (Table 5) involves interaction of gene(s) on chromosome 7 (*Glyma07g32480*) and chromosome 15 (*Glyma15g42220*), both of which show high levels of expression in RILs with extreme root phenotypes (Figure 6). Non-synonymous mutations were present in both of these genes in V71-370 and PI407162. The genes in the QTL interval region for root thickness of 0.5–1.0 mm (*Glyma08g19050*) and taproot length (*Glyma15g42220*) showed higher expression in RILs with roots smaller than the *G. soja* parent.
  Among the 18 genes selected in QTLs based on the *G. soja* IT182932 sequence (Tables 7 and 8), only three genes (Table 7) encoding *peroxidase* (two genes) and *CASP like protein* showed differential gene expression associated with the root phenotype of the parents and RILs (Figure 7B). The missense mutations in these two *peroxidase* genes (Table 9) might contribute to the higher levels of expression in *G. soja* than in *G. max*; however, this needs to be validated with further gene knockout experiments. One of the genes with a non-synonymous SNP on chromosome 6, *Glyma06g46210*, encodes a NEDD8-activating complex and showed an interesting pattern of expression in the RILs, with higher expression in one of the high (HR2) and low extreme RILs (LR3) (Figure 8). Higher gene expression in both the extreme root phenotypes (low and high) could possibly be explained by an additive nature of the loci and their interaction (Table 5) to produce a better parental root phenotype. However, other genes with deleterious mutations (Table 8) showed significantly different expression patterns between parental lines. The LR3 line showed higher expression levels for most of the mutated genes identified in this study. Even though these RILs were selected based on allelic composition in this particular QTL confidence interval, they differed substantially for allelic composition within other regions of chromosome 6 (Additional file 4: Figure S3) and at the whole genome level. Thus, the gene expression in LR3, with the smallest root size, may reflect the enrichment of the *G. max*-derived alleles at all these loci.

## Discussion

### Novel wild soybean alleles for the improvement of root system architecture

Despite the narrow genetic base of cultivated soybeans [4], previous root mapping studies [36,37] successfully used intra-specific mapping populations to map root QTLs in soybean. A recent study [38] used a mapping population developed between the cultivar Jingdou23 and a semi-wild cultivar, ZDD2315, to identify genes and their regulation that control seedling coarse root traits. In the present study, we dissected the genetic variation for both coarse and fine roots using an inter-specific soybean mapping population. Interestingly, the *G. soja* parent (with smaller roots) alleles influenced the root traits of total root length and root volume, explaining a phenotypic variation of more than 10 per cent. Similar contributions of positive alleles for root length and surface area QTLs by a phosphorus inefficient genotype parental line were reported in an earlier study of soybean [39]. Similar useful alleles for a number of agronomic traits were identified from *G. soja* accessions in previous studies [19-23]. The seedling root trait, total root length, and root volume (which is highly correlated with surface area) are highly correlated with drought and aluminum tolerance indices in soybean [40-42]. These traits determine the overall root growth rate and the plasticity of root architecture of plants, and are important for effective soil exploration to intercept nutrients, and for communication of stress cues [43]. A deeper root (influenced by total root length) is vital to improve drought tolerance and is positively correlated with yield under drought stress in both soybean [44] and rice [29,45]. The total root length/surface area plays a crucial role in foraging and accumulation of phosphorus [39], and also improves the limiting effect of phosphorus on shoot growth [46]. The QTL region on chromosome 6 identified in this study also harbors a domestication-related trait for 100-seed weight [19] and seed yield [47]. This QTL region co-locates with another root QTL identified in other studies near Satt357-Satt202. These markers flank the QTL for mean root length [37] and yield [47] that explained phenotypic variances of 26% and 8%, respectively. This region also possesses different aquaporin genes (involved in water transport) that interact with other aquaporin family members on chromosomes 8 and 12 [37].

Alleles from the *G. max* parent, V71-370, also contributed to the development of fine root structure and distribution. Studies focused on fine root structure in legume crops have used pulses [48], but none have been completed in soybean. The fine root distribution, based on length, surface area, and volume, showed high positive correlation with total root volume. The distribution based on thickness was highly correlated with surface area only. Small diameter roots reflect the proportion of fine lateral roots, which enhance the whole root surface area, acting as an exchange site between the plant and the soil [49]. In this study, several genes associated with different root architectural traits were identified, based on microarray analyses (Table 4) and whole genome sequencing analysis of two *G soja* lines IT182932 and PI 407162 (Tables 7 and 8). Most of the root traits reported in this study were associated with the expression of one or polygenes. Similar genetic regulation of root traits by major gene and polygenes was reported in soybean recently during seedling-stage development [38]. In the present study, most of the root traits showed a transgressive segregation pattern. A similar segregation pattern was reported for maximum root length and lateral root number in soybean [38]. However, tomato introgression lines developed between cultivated and wild tomatoes showed both transgressive and continuous patterns [31]. As the QTL regions identified governs both coarse and fine roots, these might be candidate regions to develop a better root ideotype in soybean.

### Genes associated with root QTLs

Among the 10 candidate genes (Table 4) identified based on transcript abundance from the microarray analysis, *kinesin* was also found to be highly expressed in maize root tissues [50]. Other genes, such as *carboxylesterase 6 like* and *histone-like CCAAT transcription factor*, were also found to be highly expressed (Figure 5) in root pericycle cells, which regulate lateral root formation. These genes were also found to be upregulated in water-deficit conditions based on an Affymetrix gene chip study (Babu et al. unpublished) of different root regions. The *carboxylesterase 6 like* gene was highly upregulated under drought stress conditions in root region 2 (4- to 8-mm tip). It was also upregulated in the root region 1 (0–4 mm) following drought stress, but at lower levels compared with region 2. The role of this gene in the maintenance of root growth during drought stress conditions has yet to be studied. Candidate genes identified in the *G. soja* line, such as *Apoptosis inhibitor 5 related* (*Glyma07g32480*), *slow anion channel associated 1-like* (*Glyma15g42220*), and *Metacaspase* (*Glyma08g19050*) (Table 4), showed high expression in root-related tissues (Figure 5) and could be key candidates to improve root length and diameter in cultivated soybean, which might increase nutrient and water use efficiency. Emphasis should be placed on characterizing the function of an uncharacterized gene, *Glyma15g42220*, which is associated with soybean root system architecture, because it showed higher expression in RILs with the shortest root length and surface area compared with RILs with the longest root length. The sequence of this gene is similar to that of *slow anion channel associated 1* in other crops, which is involved in osmoregulation, phosphorus uptake [51], and aluminum tolerance [52]. It is also reported to be involved in regulating the exchange of water vapor and gas during drought stress [53].

The genes with high levels of transcript abundance expressed only in root tissues within the QTL region of chromosomes 6 (Table 7) had distinct expression patterns in the cultivated and *G. soja* parents. Similar trends of gene expression were reported for genes related to stress, defense response, and redox pathways observed in wild soybean [54] and wild tomato [55] compared with their respective cultivated types. Among the 18 genes identified based on sequence information from a wild soybean, two *peroxidase* genes (*Glyma06g45910* and *Glyma06g45920*) in different clades of the heat map showed high expression in the *G. soja* line (Figure 7B). One of these *peroxidase* genes maps to a duplicated region [56] and showed higher expression in the RILs with small or larger root phenotypes. A similar trend of gene expression was observed in tomato introgression lines developed between cultivated and wild tomatoes [57]. This peroxidase gene in PI407162 also had a non-synonymous SNP variation that changed an amino acid from serine to alanine. Similar effects on root size of amino acid changes were reported in the phosphorylation of the ETHYLENE *INSENSITIVE2* gene in Arabidopsis [58]. Zhu et al. [59] reported that the peroxidase activity was spatiotemporal in root tissues and was involved in both cell wall loosening and tightening under water-deficit conditions. Voothuluru and Sharp [60] also reported the effect of *peroxidase* genes on apoplastic reactive oxygen species in the root apical region and its effect on cell wall modification in maize roots as a part of a drought adaptive mechanism. The single copy genes identified in the present study could also be used as informative markers for phylogenetic and comparative analyses across various taxonomic levels [61].

### Effect of non-synonymous SNP mutations on root architecture

Our results indicated that non-synonymous SNP mutations in genes underlying the QTL region on chromosome 6 are critical candidate genes to study root growth and development. In particular, we identified the gene encoding NEDD8-activating complex, a ThiF protein family (Pfam ID: 00899) that is associated with production of ubiquitin-activating enzyme E1 (Panther ID: 10953), which controls phosphate starvation responses through shoot and root modifications. This gene showed higher expression in wild soybean PI 407162 and the HR2 RIL with higher total root length and surface area. This expression pattern indicated that the transcriptional landscape of *G. soja* lines was highly diverged relative to cultivated soybean at this particular locus. The homologous gene to *NEDD8-activating complex* also showed higher expression in wild tomatoes than in cultivated tomatoes, as a result of accelerated evolution [55]. The HR2 RIL’s higher expression for this gene might have occurred as a result of the interaction between the *G. soja*-derived mutated allele (containing five non-synonymous SNPs within the coding region) and the *G. max*-derived allele and para-mutating its effect. Similar mechanisms underlying the gene expression levels were observed in a maize inter-mated mapping population [62]. Thus, this transcript with high expression from wild soybeans is a candidate to increase root length through coordinating cell division and elongation dynamics, as has been reported in wild tomatoes [31]. The TPR transcription factor, with a non-synonymous mutation, was identified among a number of *G. soja* accessions (Table 10) and showed similar expression patterns in both parental lines and RILs. A similar class of transcription factor was reported to be expressed in roots as an early response to iron availability in soybeans [63]. Mutations of key genes with insertions or deletions associated with rate of cell divisions (*D6 type cyclin* and *auxin efflux carrier protein*) might result in the shorter root phenotypes in *G. soja*, PI407162, compared with V71-370. Sequences derived from other *G. soja* accessions [8,35] also showed the same non-synonymous SNP in these same genes. As a result, the altered protein products of these two genes might have resulted in shooter root phenotypes in all wild soybeans. Similar molecules were reported in Arabidopsis and rice to influence cortex-endodermis division regulated by the *SHORT-ROOT* transcription factor [64,65].

### Comparative genomic analysis among *G. soja* accessions

Based on the whole genome re-sequence, information from 17 accessions, which represent diverse *G. soja* accessions from Korea [8] and China [35], also showed conserved non-synonymous SNPs in most of the root related genes identified in this study (Table 10). Among different plant species, soybeans are reported to have a higher average ratio of Nonsyn/Syn SNPs than Arabidopsis and rice, with larger effect SNPs in 4,648 genes that have greater effects on their functions [35]. In addition, 21% of potential SNP loci were fixed during the domestication process from wild soybeans. Therefore, the genes identified in the present study might represent candidates with high biological significance for root system growth and development. These genes may play a key role in maintaining short root phenotypes of wild soybeans. A strong candidate for further study is the auxin responsive gene, *NEDD8-activating complex,* because it influences the total root length and involves a key hormone, auxin, which regulates the root growth and development. In Arabidopsis, this gene interacts with a *CULLIN* gene, which then alters the growth of lateral roots and root hairs [66]. Similar results were observed in elite lines of rice in which SNPs altered the expression of *9-cis-epoxycarotenoid dioxygenase* gene associated with the ABA content. A rice line with this missense mutation had produced more lateral roots [67]. For the uncharacterized gene (*Glyma06g46170*), a gain of a stop codon in the *G. soja* PI 407162 makes this a potential candidate gene to further study the functional effects associated with root development in soybeans. Therefore, the root-related genes with non-synonymous mutations identified in this study are a valuable genetic resource to study the evolution of root system development in soybeans. The candidate genes identified from the *G. soja* accession, PI 407162, are promising targets to improve root system architecture in cultivated soybeans. However, RILs with soja alleles show undesirable phenotypes, such as lodging and poor yield, which could be eliminated by a series of backcrosses with the cultivated soybean, V71-370.

## Conclusion

The QTL regions on chromosome 6 for total root length and root volume, and the QTLs on chromosome 7 for finer roots will enable us to integrate improvements in root architecture in soybean. The candidate genes associated with root traits, and with non-synonymous mutations are valuable genetic resources for understanding the evolution of the longer root phenotype in cultivated soybeans compared with the short roots found in most *G. soja* accessions. The genes identified in this study will be important for understanding the molecular mechanisms and gene regulation associated with root development. These findings also suggest that combining novel rare alleles from wild soybean with those of cultivated soybeans could be used to modify/alter the RSA of cultivated soybeans and also develop a soybean that is suited to soils with varying nutrient and water availabilities.

## Methods

### Plant materials

A subset of 160 F_12_ recombinant inbred lines of a mapping population derived from a soybean inter-specific cross between *G. max* (long and robust root system) × *G. soja* (smaller roots) was selected to map QTLs for root system architectural traits. The population was first developed to map resistance genes for *Phytophthora sojae* in soybean [68,69]*.* However, in the seedling stage the parental lines also differ for various components of the root system architecture, which enabled us to map QTLs for various root traits in the present study.

### Plant growth conditions and root tissue sampling

Parental lines and the RILs were grown in a cone system, replicated four times in a completely randomized block design, using DL60L cones and D20 supporting racks (Stuwe and Sons, Oregon, USA). Each replication was conducted separately in the Sears Greenhouse Facility, University of Missouri, USA, from September 2011 to December 2012. Turface (Turface Athletics, Illinois, USA) and sand was mixed in a 1:1 ratio as a growing medium in cones to offer mechanical impedance similar to field conditions and to facilitate removal of the root system without damage. The day and night temperature in the greenhouse were maintained at 29°C and 21°C, respectively. The photoperiod was set at 12 h using overhead 400 W metal halide lamps that generated a photosynthetic photon flux density of approximately 1620 μmol m^−2^ s^−1^. The seedlings were grown up to V1 growth stage (approximately 14 days after sowing) and the intact seedlings from the cones were collected and analyzed [70].

### Phenotypic data

Root samples were transferred into water-filled clear trays to carefully remove turface particles firmly attached to the root. The roots were then transferred into another water-filled tray, scanned using an Epson Scanner 10000XL (Epson America Inc., CA, USA) and analyzed using WinRhizo software (Regent Instruments Inc.,Canada). In addition to manual measurements of taproot length and root fresh weight, data on total root length, surface area, average diameter, root volume, lateral average diameter, tertiary root number, tertiary root length, and root distribution classification based on length, surface area, volume, and thickness were derived from the imaging analysis. The Proc General Linear Model (GLM) and analysis of variance analysis was performed using SAS (v. 9.3).

### Genotypic data

The total RNA isolation and Affymetrix microarray data analysis and data processing were performed at the Core Laboratory Facility, Virginia Bioinformatics Institute, Virginia Tech, as described in Zhou et al. [32]. The algorithms used to identify informative SFP markers are explained in Additional file 5: File S1, Figures S4-S7. The SFP marker data generated were combined with 109 publicly available simple sequence repeat (SSR) markers that span all linkage groups, and used to construct a genetic linkage map with the Kosambi mapping function in JoinMap 3.0 [71] at the University of Missouri, Columbia. The initial linkage grouping of markers were performed with a likelihood of odds (LOD) threshold score of 3.0 and a maximum genetic distance of 50 cM. The chromosomes were numbered [72] corresponding to the designated soybean genetic linkage groups [73].

### QTL mapping analysis

A high-density genetic map was created with 1,046 molecular markers that included 937 SFPs and 109 SSRs, with an average marker separation of less than 2 cM. The interval mapping (IM) method was conducted to predict QTLs, followed by composite interval mapping (CIM) using Qgene v4.3.6 [74], with permutations of 1000 iterations to declare the QTL as significant. For CIM, stepwise cofactor selection was used with markers as cofactors and the maximum number of cofactors was selected automatically (F to add = 0.01 and F to drop = 0.01). The permutation LOD value at p ≤ 0.05 was used as the threshold to declare the significance of the QTLs. The interactions among QTLs were identified using QTLNetwork-v2.1 software, with a mixed linear model based on CIM with a 10 cM window size and 1 cM walking speed. Permutation tests with 1,000 runs were used to determine the threshold of the F-value for the significance of QTLs to control genome-wide type I errors. Digenic interactions were also analyzed using a mixed-model approach. Significance levels for the genome scans for candidate intervals, QTL detection, and effects were set at 0.05, 0.001, and 0.001, respectively. The epistatic interaction between QTLs was illustrated using the Circos software [75].

### Candidate gene selection for real-time quantitative reverse-transcription PCR (qRT-PCR) analysis

#### Based on transcript abundance in microarray analysis

For each root QTL identified, all the genes (Table 4) that were located between the flanking markers were examined for their presence on the Affymetrix chip, as well as for changes in transcript abundance. The genes were annotated as described in [32] and are listed in Table 4. The microarray data generated in this mapping population, along with the RILs [32], are available at http://www.ncbi.nlm.nih.gov/geo/query/acc.cgi?acc=GSE11611 in the NCBI database. The primers sequences for the genes selected based on transcript abundance are shown in Additional file 6: Table S3.

#### Based on wild soybean sequencing information

To identify the genes specific to the wild soybean at the QTL interval on chromosome 6 flanked by the Affymetrix probe sets 4222.1.S1_10 and 77599.1.S1_7, DNA sequencing information of a *G. soja* variety, IT182932 [8] was used. Based on the analysis, 162 genes were identified within the QTL interval region based on the SSR marker position in linkage map (Satt316; 126 cM, 47.5 Mb to Satt357; 143.6 cM, 49.8 Mb). These SSR markers flanked the Affymetrix probe sets. For these genes, BLAST analysis was performed using the BLASTX algorithm (E ≤ 1e-6) against the non-redundant protein NCBI database and annotated using Blast2GO software [76]. The number of genes were narrowed down to 18 genes (includes nine genes with non-synonymous SNPs) with high transcript abundance in root tissues (Tables 7 and 8), based on public soybean RNA sequencing data available in SoyKB [33]. The primers designed for the genes selected based on transcript abundance in microarrays [32] are shown in Additional file 7: Table S4, and genes with missense mutations are shown in Additional file 8: Table S5.

### RNA isolation and qRT-PCR

RNA was extracted from root tissues (100 mg tissues) collected from parental lines (V71-370 and PI407162) and selected RILs using an RNeasy Plant mini kit (Qiagen, CA, USA), according to the manufacturer’s protocol. On-Column DNA digestion was performed using RNase-Free DNase Set (Qiagen), according to the manufacturer’s protocol. Each sample (2 μg of total RNA) was reverse transcribed to cDNA in a 20-μL reaction volume using RNA to cDNA EcopryTM Premix (Double primed) cDNA Synthesis Kit (Clontech, CA, USA). The qRT-PCR was performed using the cDNA product corresponding to 25 ng of total RNA in a 10-μL reaction volume and Maxima SYBR Green/ROX qPCR Master Mix (2×) (Thermo, USA) on a ABI7900HT detection system (Applied BioSystems, Foster City, CA, USA). The expression data for each sample were generated from three biological and two technical replicates. The relative expression of the selected genes were expressed as the mean standard deviation, in comparison with the transcript abundance of actin, a housekeeping gene, and analyzed using the Delta Ct method [77]. The PCR conditions were as follow: 50°C for 2 min, 95°C for 10 min, then 40 cycles of 95°C for 15 s, 60°C for 1 min. To normalize the gene expression, Actin (*Glyma18g52780*) was used as an internal control. All primers were designed using the Primer3 web-interface (http://frodo.wi.mit.edu/primer3/input.htm) [78]. Gene expression was evaluated among parental lines and selected RILs of extreme phenotypes (Additional file 9: Table S2). The chromosome graphical representation of the selected RILs was made with graphical genotype software GGT (v 2.0) [79].

### DNA isolation, genome sequencing, and functional SNP identification

The DNA of the parental lines (V71-370 and PI407162) was isolated and sequenced at a depth of 15× using Illumina 90 bp paired-end sequencing technology with insert sizes of around 500 bp. The data were processed after filtering out low-quality reads and duplicate reads. The processed data were aligned to the William 82 *G. max* v1.1 from Phytozome as the reference genome [2]. SNPs and Indels were identified using an in-house built pipeline using GATK v3.0 [80] and were analyzed for possible synonymous/non-synonymous SNP variation annotations using SnpEFF [81] and v9.0 gene models from Phytozome. To detect small insertions and deletions, Indels (1-5 bp) were called by SOAP (Short Oligonucleotide Analysis Package) indel 1.09 (http://soap.genomics.org.cn/soapindel.html). The non-synonymous SNP variations were only considered for comparison among 17 Chinese and 1 Korean wild soybeans, and can be viewed using SNPViz tool [82] available in SoyKB.

### Availability of supporting data

All the supporting data are included as additional files.

## Additional files

Additional file 1: Figure S1. Histogram of the frequency distribution of root traits among RI lines of the mapping population (V71-370/PI407162). Additional file 2: Table S1. List of other root trait QTLs co-located on chromosome 6 and 7. Additional file 3: Figure S2. Pie chart representation of the ontological classification of genes underlying the candidate QTL region on chromosome 6. Additional file 4: Figure S3. Genomic compositions of selected extreme RILs on chromosome 6 (three High Roots (HR) lines denoted as 1–3, and three Low Roots (LR) lines as 4–6) for gene expression study using qRT-PCR. Additional file 5: File S1. The SFPdev Min-Max Ratio algorithm. Figures S4-S7: Algorithms used for polymorphic single feature polymorphism (SFP) detection in Additional file 5: File S1. Additional file 6: Table S3. The primer sequences for genes with high transcript abundance within the QTLs identified. Additional file 7: Table S4. The primer sequence for genes selected based on wild soybean variety IT 182932 within the QTL interval on chromosome 6. Additional file 8: Table S5. The primer sequences for genes with a deleterious mutation based on wild soybean variety IT 182932 within the QTL region on chromosome 6. Additional file 9: Table S2. Extreme root phenotypic RI lines selected for qRT-PCR gene expression analysis.
