# Supplementary figures and images for: Genetic variants in root architecture-related genes in a Glycine soja accession, a potential resource to improve cultivated soybean
Source: BMC Genomics. 2015 Feb 25;16(1):132. doi: 10.1186/s12864-015-1334-6 (PMC4354765; doi:10.1186/s12864-015-1334-6)

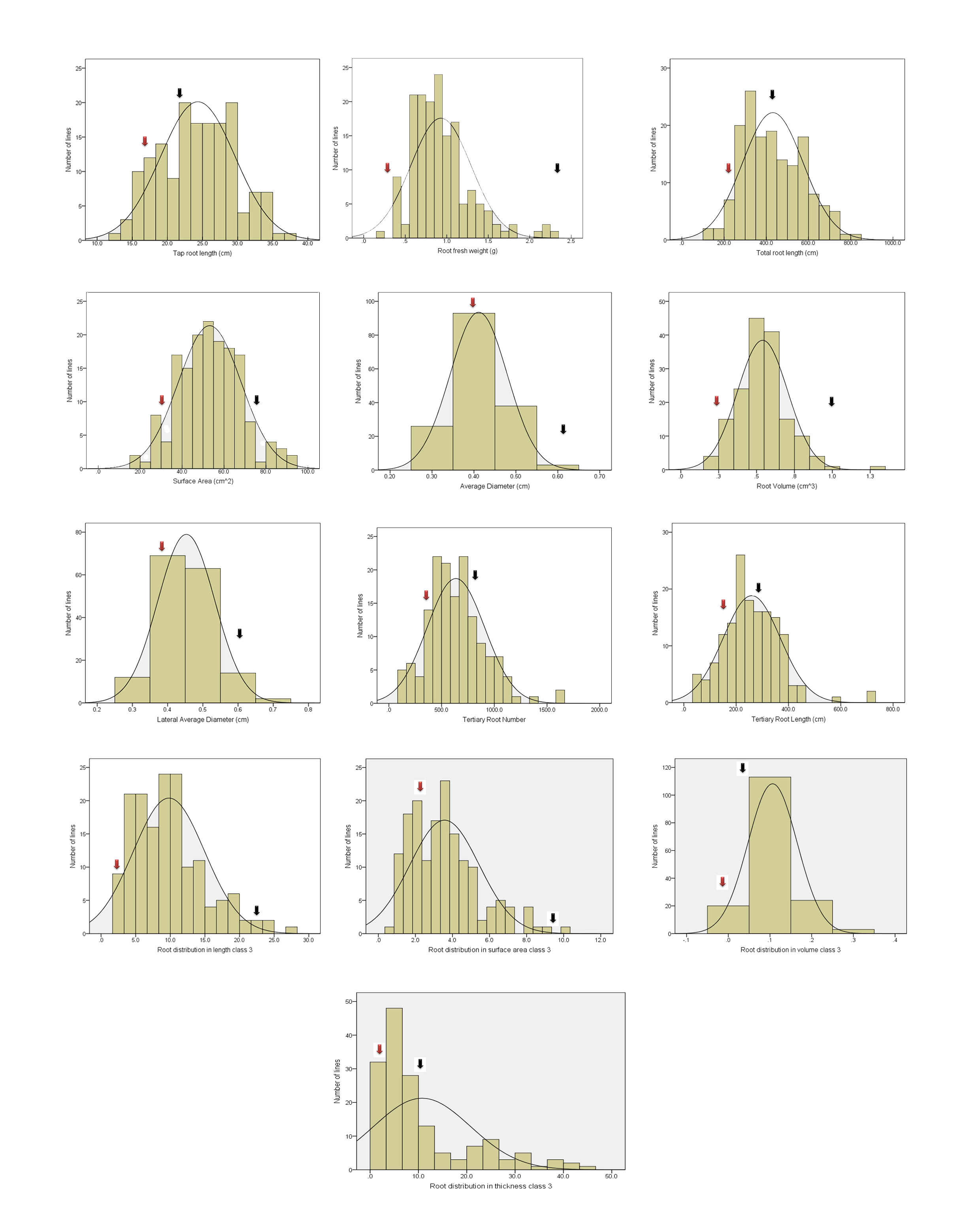

Supplement: Additional file 1: Figure S1. — Histogram of the frequency distribution of root traits among RI lines of the mapping population (V71-370/PI407162). [file 12864_2015_1334_MOESM1_ESM.png]

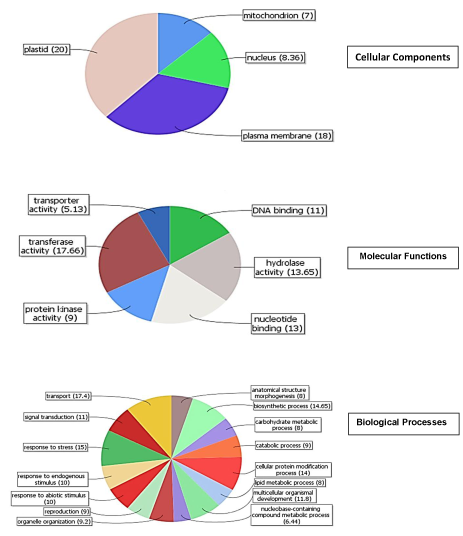

Supplement: Additional file 3: Figure S2. — Pie chart representation of the ontological classification of genes underlying the candidate QTL region on chromosome 6. [file 12864_2015_1334_MOESM3_ESM.png]

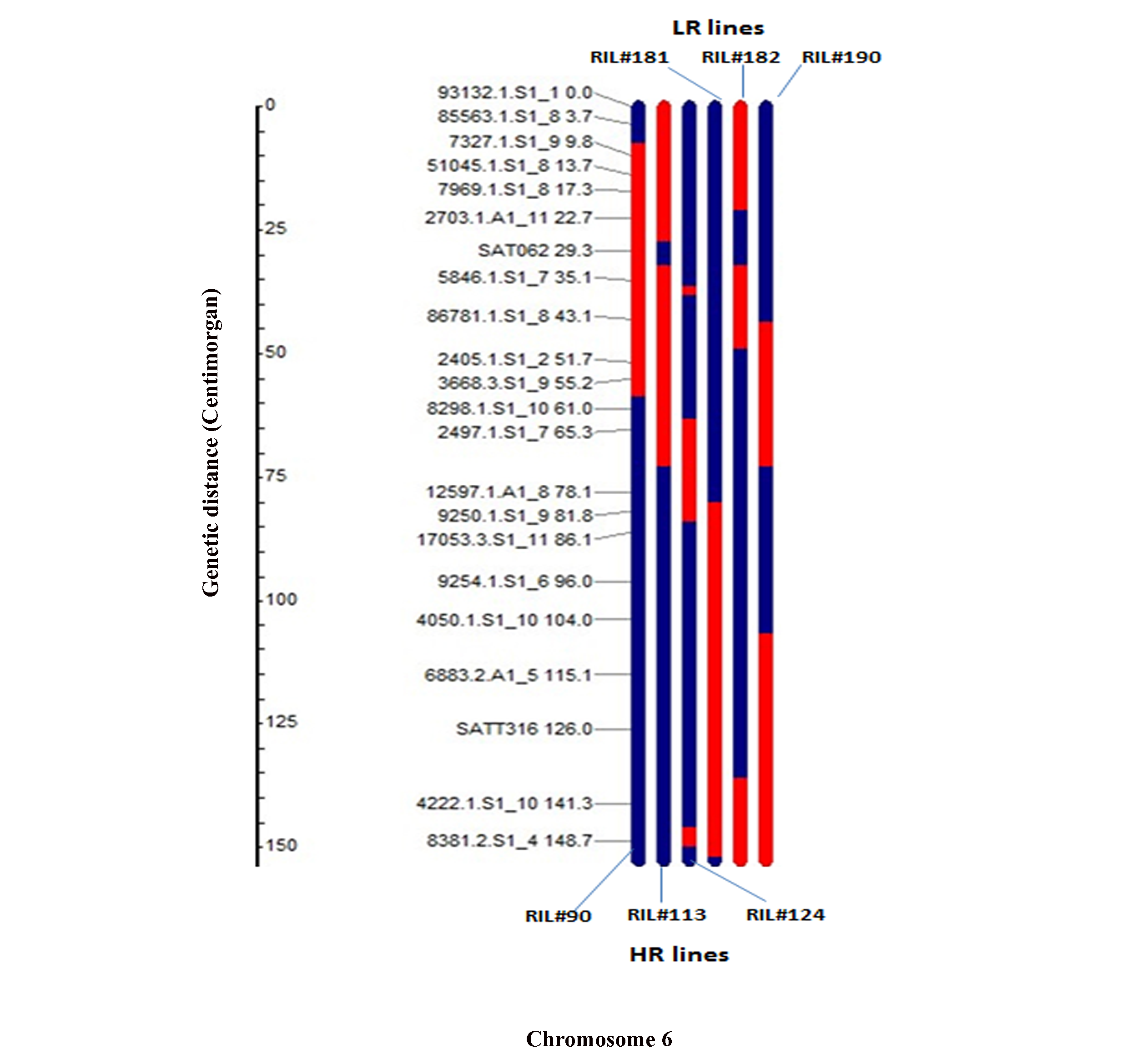

Supplement: Additional file 4: Figure S3. — Genomic compositions of selected extreme RILs on chromosome 6 (three High Roots (HR) lines denoted as 1–3, and three Low Roots (LR) lines as 4–6) for gene expression study using qRT-PCR. [file 12864_2015_1334_MOESM4_ESM.png]
