# Supplementary material for: Genetic variants in root architecture-related genes in a Glycine soja accession, a potential resource to improve cultivated soybean
Source: BMC Genomics. 2015 Feb 25;16(1):132. doi: 10.1186/s12864-015-1334-6 (PMC4354765; doi:10.1186/s12864-015-1334-6)
Supplement: Additional file 2: Table S1. — List of other root trait QTLs co-located on chromosome 6 and 7. [file 12864_2015_1334_MOESM2_ESM.docx]

Additional Table 1. List of other root trait QTLs co-located on chromosome 6 and 7

| **S.No** | **Trait** | **Chr.** | **LG** | **Marker interval** | **LOD value** | **R2 value** | **Additive effect** |
| --- | --- | --- | --- | --- | --- | --- | --- |
| 1 | RV | 6 | C2 | 4222.1.S1_10 - 77599.1.S1_7 | 3.2 | 7.8 | -0.05 |
| 2 | TRTL | 6 | C2 | SATT357 - 8381.2.S1_4 | 3.9 | 6.9 | -1.5 |
| 3 | TERL | 6 | C2 | 4222.1.S1_10 - 77599.1.S1_7 | 3.4 | 9.5 | -32.2 |
| 4 | RDSA3 | 7 | M | 8398.1.S1_11 - 1900.1.S1_3 | 3.6 | 9.8 | 0.59 |
| 5 | RDV3 | 7 | M | 59884.1.S1_8- 8398.1.S1_11 | 3.6 | 9.8 | 0.02 |
