# Supplementary material for: Genetic variants in root architecture-related genes in a Glycine soja accession, a potential resource to improve cultivated soybean
Source: BMC Genomics. 2015 Feb 25;16(1):132. doi: 10.1186/s12864-015-1334-6 (PMC4354765; doi:10.1186/s12864-015-1334-6)
Supplement: Additional file 5: File S1. — The SFPdev Min-Max Ratio algorithm. Figures S4-S7: Algorithms used for polymorphic single feature polymorphism (SFP) detection in Additional file 5: File S1. [file 12864_2015_1334_MOESM5_ESM.docx]

Additional File 5

SFPdev Min-Max Ratio algorithm

This algorithm (Additional Figure 4) first calculates the SFPdev statistic, which is the absolute difference of hybridization intensity value of each probe from the average of the probeset, divided by the value for that probe. Each probeset corresponds to a gene and is composed of 11 PerfectMatch (PM) probes, and 11 MisMatch (MM) probes (details at <http://www.affymetrix.com>). The MM probes are not included in the calculation. The SFPdev values are calculated for each of the four replicate microarrays, and their distribution across the replicates is also computed. The SFPdev value is higher in the case of a polymorphic probe, since the reduced hybridization results in greater deviation from the average intensity of the probe set (Additional Figure 4). The calculation of this statistic is repeated for each of the RILs separately. Then by comparing pairs of RILs *a*,*b* (Additional Figure 5) we accept a probe as having a SFP polymorphism, if the ratio of the smallest value SFPdev_a_ in the distribution of values from RIL a carrying the polymorphism, divided by the largest SFPdev_b_ from RIL b (or vice versa) is greater than two-fold. This is an empirical threshold reported during the first implementation of the algorithm (West et al. 2006), and also verified while applying this algorithm to our data.

Additional Figure 4. SFP discovery with Affymetrix probes


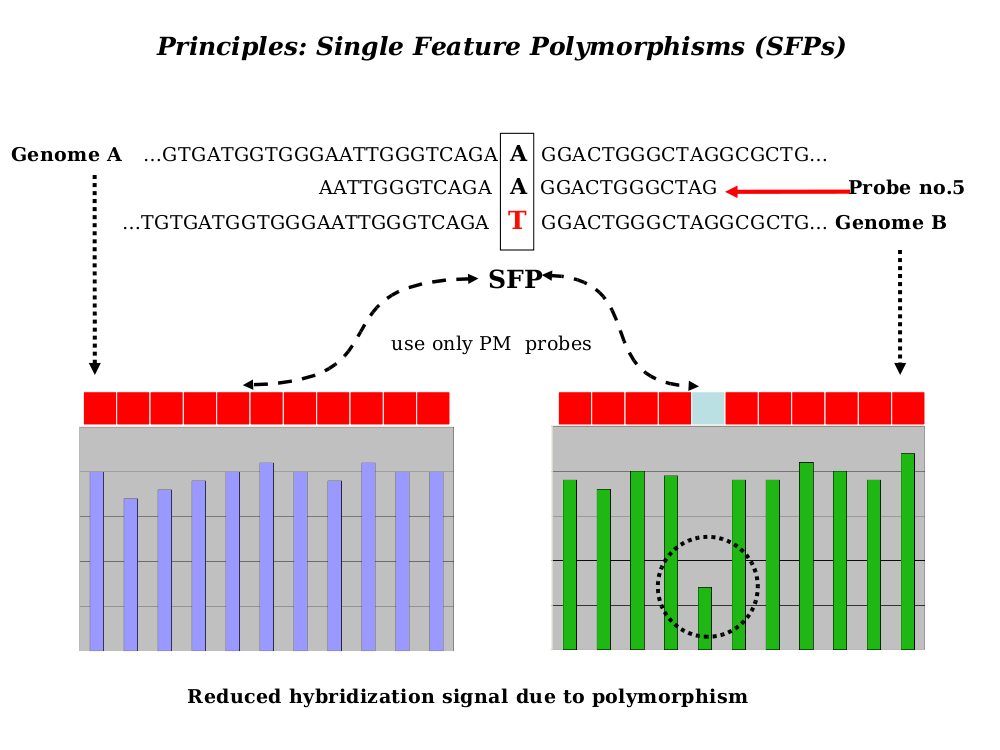


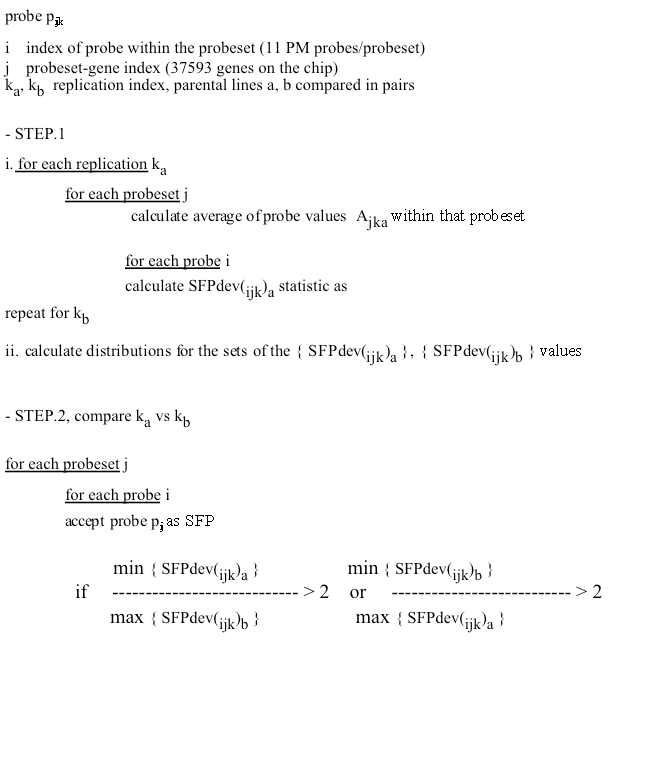


Additional Figure 5. SFPdev Min-Max Ratio algorithm

RIL Bimodal Distributions algorithm. This algorithm is similar to K-means clustering with K = 2. In summary, the RIL Bimodal Distributions (Additional Figure 6) algorithm first calculates the absolute values of probe intensity differences d_ijk_ (probe i, probeset j, RIL k), from the average of each probeset. Similarly with the SFPdev, only PM probes are included in the calculation. In the next step, the algorithm computes the distribution of each d_ijk_ value across all the individuals of the RIL population. The median M_ij_ is initially used to split the distribution into an upper (u) and lower (l) subsets (Additional Figure 7). The averages l_avg_ , u_avg_ of the l and u subsets respectively, are the seeding centers for the K-means clustering. Then the algorithm iterates in the same manner for eight times , but instead of the M_ij_, it uses the average (l_avg_ + u_avg_)/2 for splitting again into u,l subsets. After all iterations, the d_ijk_ values settle into a bimodal distribution, with each mode corresponding to a K-means cluster. These steps are repeated for probes in all the probesets, measured in the expression profile of each individual of the population. In order to assess significant separation between the two distribution modes (or otherwise the two clusters), we use as metric the peak separation ps = (A_l_ – A_u_)/√(S_l_ ^2^/n_l_ + S_u_^2^/n_u_) (A_l_ and A_u_ are distribution averages for the u,l modes respectively, standard deviations S_l_ and S_u_, sample sizes n_l_ and n_u_).

The algorithm also computes the d_ij_ values (averaged across replicate microarrays) for the PI407162 and V71-370 parental data. For polymorphic probes, d_ijk_ values for the individuals of the RIL population are expected to cluster around the parental d_ij_, under the two modes of the distribution (Additional Figure 7). Since the RIL population was created by the cross of the genetically distant PI407162 and V71-370 soybean lines, the two modes originate due to the different parental alleles inherited to the RIL progeny. For each SFP probe, RIL individuals are assigned a genotype based on their clustering around one of the parental values (Additional Figure 7).

Additional Figure 6. Details of the RIL Bimodal Distributions algorithm


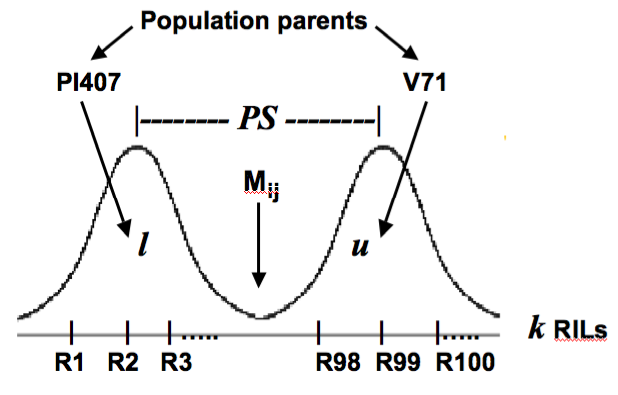


PI407162

V71-370

Additional Figure 7. Genotyping RILs based on parental genotypes to the bimodal distribution
