# Supplementary material for: Genetic variants in root architecture-related genes in a Glycine soja accession, a potential resource to improve cultivated soybean
Source: BMC Genomics. 2015 Feb 25;16(1):132. doi: 10.1186/s12864-015-1334-6 (PMC4354765; doi:10.1186/s12864-015-1334-6)
Supplement: Additional file 6: Table S3. — The primer sequences for genes with high transcript abundance within the QTLs identified. [file 12864_2015_1334_MOESM6_ESM.docx]

| **S.No** | **Gene ID** | **Sequence 5`---------3`** |
| --- | --- | --- |
| 1 | Glyma06g46680-qPCR-F | ACGGAGATGGAGTACTATGAGG |
|  | Glyma06g46680-qPCR-R | TGAGAGCATCGGTTTGAGC |
| 2 | Glyma06g46850-qPCR-F | AGAGAAGGAAAGCTGTTGGTG |
|  | Glyma06g46850-qPCR-R | CAGCTCACGCATCTTACTCC |
| 3 | Glyma07g09530-qPCR-F | GGCTCTTCAGTCTTATCTCGTG |
|  | Glyma07g09530-qPCR-R | ACGGTCTACATTGTTTGGAGG |
| 4 | Glyma09g32280-qPCR-F | TGGTTACACTCTTCGGCTTAC |
|  | Glyma09g32280-qPCR-R | CTCTCGCCGTGTTTGATTTTC |
| 5 | Glyma07g09860-qPCR-F | TCGTGTTGCTCTCCATCTTG |
|  | Glyma07g09860-qPCR-R | ACCCAAGTCCAAAGGCATAG |
| 6 | Glyma09g31950-qPCR-F | TCGTGTTGCTCTCCATCTTG |
|  | Glyma09g31950-qPCR-R | ACCCAAGTCCAAAGGCATAG |
| 7 | Glyma07g11880-qPCR-F | AACCTACGCCCATTCAATCTC |
|  | Glyma07g11880-qPCR-R | AGTAAGCAAGTGTCTTCCCTG |
| 8 | Glyma08g20670-qPCR-F | CTTTCACGATGCTGGTTTTCC |
|  | Glyma08g20670-qPCR-R | GTCTTCCCTGATCCTGTTTCTG |
| 9 | Glyma07g01260-qPCR-F | AACCTACGCCCATTCAATCTC |
|  | Glyma07g01260-qPCR-R | AGTAAGCAAGTGTCTTCCCTG |
| 10 | Glyma07g32480-qPCR-F | CCAGGGAATCATAGATGCGG |
|  | Glyma07g32480-qPCR-R | GGGCTAGAAAGGTCAGGAAAG |
| 11 | Glyma13g24090-qPCR-F | CCAGGGAATCATAGATGCGG |
|  | Glyma13g24090-qPCR-R | CCTCAACCAAATCAAGATGCG |
| 12 | Glyma02g15190-qPCR-F | CGAGAACTACCTCCACAACTG |
|  | Glyma02g15190-qPCR-R | GAACTCCACGAATCCATATCCC |
| 13 | Glyma07g33300-qPCR-F | CGAGAACTACCTCCACAACTG |
|  | Glyma07g33300-qPCR-R | GAACTCCACGAATCCATATCCC |
| 14 | Glyma15g41600-qPCR-F | CCTGCAAACCCAAAGATTCAC |
|  | Glyma15g41600-qPCR-R | TCCCTGCACCTGTTATTGTAC |
| 15 | Glyma15g42220-qPCR-F | AAATTCTCAACCTGCTCTCCC |
|  | Glyma15g42220-qPCR-R | TCACTTGGTCCATGAAGCTC |
| 16 | Glyma04g42630-qPCR-F | TGAAGATATGCCCTCCGAATG |
|  | Glyma04g42630-qPCR-R | GTCAGCGTGTATAGCCTCATC |
| 17 | Glyma06g12140-qPCR-F | GATGAGGCTATACATGCTGACC |
|  | Glyma06g12140-qPCR-R | GAAACATGCTCTGGAACACTG |
| 18 | Glyma11g26970-qPCR-F | AAGATTCAGTCCAAGCACCTC |
|  | Glyma11g26970-qPCR-R | CGGGCTAGTAAGATCATGGTTG |
| 19 | Glyma18g07050-qPCR-F | GATCTTACTAGCCCGGTGAAG |
|  | Glyma18g07050-qPCR-R | CCAACTGACCCTGACCTAATATG |
| 20 | Glyma08g19100-qPCR-F | AGGGACGGAAACAAAAGGAAG |
|  | Glyma08g19100-qPCR-R | AACCCTCGTTAGATGCCTTG |
| 21 | Glyma15g05900-qPCR-F | AGGGACGGAAACAAAAGGAAG |
|  | Glyma15g05900-qPCR-R | ACCCTTGTTAGATGCCTTGG |
| 22 | Glyma08g19050-qPCR-F | TGGTTACAAAGGCACAAGAGG |
|  | Glyma08g19050-qPCR-R | GCATGGCTTGGATGAAACTG |
| 23 | Glyma09g28430-qPCR-F | ATCAGGCTGTAAATGTGGGAG |
|  | Glyma09g28430-qPCR-R | CAATGACAAACTCCCCAAGC |

Additional Table 3. The primer sequence for genes with high transcript abundance within the QTLs identified
