# Supplementary material for: Genetic variants in root architecture-related genes in a Glycine soja accession, a potential resource to improve cultivated soybean
Source: BMC Genomics. 2015 Feb 25;16(1):132. doi: 10.1186/s12864-015-1334-6 (PMC4354765; doi:10.1186/s12864-015-1334-6)
Supplement: Additional file 7: Table S4. — The primer sequence for genes selected based on wild soybean variety IT 182932 within the QTL interval on chromosome 6. [file 12864_2015_1334_MOESM7_ESM.docx]

Additional Table 4. The primer sequence for genes selected based on wild soybean variety IT 182932 within the QTL interval on chromosome 6

| **S.No** | **Gene details** | **Sequence Information** |
| --- | --- | --- |
| 1 | Glyma06g44980.1-qRT-F | CAGGATATCTTACAGCTGCCG |
|  | Glyma06g44980.1-qRT-R | GCCACCTAGTAATGTAGAAGCC |
| 2 | Glyma06g44880.2-qRT-F | TGAATCTCACGAATAAGGACGG |
|  | Glyma06g44880.2-qRT-R | GATTTACAGGGACTCCAGCTC |
| 3 | Glyma06g44900.2-qRT-F | GAGGTGTATACGGTCCTGATG |
|  | Glyma06g44900.2-qRT-R | GCCATCAACATAAGTCCACATG |
| 4 | Glyma06g45910.2-qRT-F | CAACAGGACGAAGGGATGG |
|  | Glyma06g45910.2-qRT-R | GAGTGTTAGTTGGGTAGTGAGG |
| 5 | Glyma06g45920.1-qRT-F | GAAGAGGCATTGCTTAGCTTG |
|  | Glyma06g45920.1-qRT-R | GTCCAACATTGCCAAAGAGTG |
| 6 | Glyma06g45980.2-qRT-F | AAGAGCCAAACAGATATCAGAGG |
|  | Glyma06g45980.2-qRT-R | AGCTGGATTCAAGTCTCTAACC |
| 7 | Glyma06g45261.1-qRT-F | ACCTTATGTGCTAGTGATGCAG |
|  | Glyma06g45261.1-qRT-R | CAGCACTATATGTCCACAAGAGG |
| 8 | Glyma06g45810.1-qRT-F | GCCTTGGATTTGCTGTTCAC |
|  | Glyma06g45810.1-qRT-R | CCTGCATCACTGTTTCCTTTC |
| 9 | Glyma06g46170.1-qRT-F | GGCACTTTCATTGTCCAAGTTG |
|  | Glyma06g46170.1-qRT-R | CCACTCTAATCCTCACACTTCTAG |
