# Supplementary material for: Genetic variants in root architecture-related genes in a Glycine soja accession, a potential resource to improve cultivated soybean
Source: BMC Genomics. 2015 Feb 25;16(1):132. doi: 10.1186/s12864-015-1334-6 (PMC4354765; doi:10.1186/s12864-015-1334-6)
Supplement: Additional file 8: Table S5. — The primer sequences for genes with a deleterious mutation based on wild soybean variety IT 182932 within the QTL region on chromosome 6. [file 12864_2015_1334_MOESM8_ESM.docx]

Additional Table 5. The primer sequences for genes with a deleterious mutation based on wild soybean variety IT 182932 within the QTL region on chromosome 6

| **S.No** | **Gene details** | **Sequence Information** |
| --- | --- | --- |
| 1 | Glyma06g45510.2-qPCR-F | CACCGTTGCGAATACTATTGC |
|  | Glyma06g45510.2-qPCR-R | CTCTTTGGCATGGCTGAATTG |
| 2 | Glyma06g45610.1-qPCR-F | AATCACGTCTACTCATGCTCG |
|  | Glyma06g45610.1-qPCR-R | CCAAGTTCAATGCTTTGTCGG |
| 3 | Glyma06g45740.2-qPCR-F | AGTAATGCGGAGCCAGATTG |
|  | Glyma06g45740.2-qPCR-R | GCATAGATCTGACAGTCCTTGG |
| 4 | Glyma06g45850.1-qPCR-F | TGCAAGGGTCTTTGGTAACC |
|  | Glyma06g45850.1-qPCR-R | CTTCTCATCCTTGGGTTACTGG |
| 5 | Glyma06g45890.1-qPCR-F | AAGTGGCGAGTGAGAATAGC |
|  | Glyma06g45890.1-qPCR-R | GAGACAACTGGTTTAGGAGATGG |
| 6 | Glyma06g46210.1-qPCR-F | CCAAACCTACCCGAGTTACAG |
|  | Glyma06g46210.1-qPCR-R | TGCACTGTCAAACTGTCCTG |
| 7 | Glyma06g46490.1-qPCR-F | AGCCATGTCATCCTTATCACC |
|  | Glyma06g46490.1-qPCR-R | CAAAGATCATGCCAGGTTTCC |
| 8 | Glyma06g46710.2-qPCR-F | ATGGATGAATGGAGGCAGAAG |
|  | Glyma06g46710.2-qPCR-R | CTGGAGTGGAAGGCAAAGAA |
| 9 | Glyma06g46730.1-qPCR-F | GTTTGATCGAGTTCCAAGAGAATG |
|  | Glyma06g46730.1-qPCR-R | ACGACACAAAGGGCAAGTAG |
