# Supplementary material for: Genetic variants in root architecture-related genes in a Glycine soja accession, a potential resource to improve cultivated soybean
Source: BMC Genomics. 2015 Feb 25;16(1):132. doi: 10.1186/s12864-015-1334-6 (PMC4354765; doi:10.1186/s12864-015-1334-6)
Supplement: Additional file 9: Table S2. — Extreme root phenotypic RI lines selected for qRT-PCR gene expression analysis. [file 12864_2015_1334_MOESM9_ESM.docx]

Additional Table 2: Extreme root phenotypic RI lines selected for qRT-PCR gene expression analysis

| **RIL No** | **Extreme root phenotype** | **SA** | **TRL** | **Allelic information in QTL** | |
| --- | --- | --- | --- | --- | --- |
|  |  |  |  | **Region on**  **Chr# 6** | **Region on**  **Chr# 7** |
| 90 | HR1 | 92.3 | 741.0 | *G. soja* | *G. max* |
| 113 | HR2 | 73.9 | 614.1 | *G. soja* | *G. max* |
| 124 | HR3 | 68.5 | 607.1 | *G. soja* | *G. soja* |
| 181 | LR1 | 18.6 | 142.8 | *G. max* | *G. soja* |
| 182 | LR2 | 26.0 | 211.0 | *G. max* | *G. soja* |
| 190 | LR3 | 16.3 | 123.4 | *G. max* | *G. max* |

HR, Lines with higher phenotypic value for selected trait; LR, Lines with low phenotypic value for selected trait; SA Surface area (cm2); TRL Total root length (cm)
